# Lipoarabinomannan biosynthesis in *Corynebacterineae*: the interplay of two α(1→2)-mannopyranosyltransferases MptC and MptD in mannan branching

**DOI:** 10.1111/j.1365-2958.2011.07640.x

**Published:** 2011-06

**Authors:** Arun K Mishra, Karin Krumbach, Doris Rittmann, Ben Appelmelk, Vibha Pathak, Ashish K Pathak, Jerome Nigou, Jeroen Geurtsen, Lothar Eggeling, Gurdyal S Besra

**Affiliations:** 1 School of Biosciences, University of BirminghamEdgbaston, Birmingham, B15 2TT, UK; 2 Institute for Biotechnology 1, Research Centre JuelichD-52425 Juelich, Germany; 3 Department of Medical Microbiology and Infection Control, VU University Medical Center1081 BT Amsterdam, The Netherlands; 4 Organic Chemistry Department, Drug Discovery Division, Southern Research Institute2000 9th Ave South, Birmingham, AL, USA; 5 Département Mécanismes Moléculaires des Infections Mycobactériennes, Institut de Pharmacologie et de Biologie Structurale, Centre National de la Recherche Scientifique Unité Mixte de Recherche 508931077 Toulouse cedex 4, France

## Abstract

Lipomannan (LM) and lipoarabinomannan (LAM) are key *Corynebacterineae* glycoconjugates that are integral components of the mycobacterial cell wall, and are potent immunomodulators during infection. LAM is a complex heteropolysaccharide synthesized by an array of essential glycosyltransferase family C (GT-C) members, which represent potential drug targets. Herein, we have identified and characterized two open reading frames from *Corynebacterium glutamicum* that encode for putative GT-Cs. Deletion of *NCgl2100* and *NCgl2097* in *C. glutamicum* demonstrated their role in the biosynthesis of the branching α(1→2)-Man*p* residues found in LM and LAM. In addition, utilizing a chemically defined nonasaccharide acceptor, azidoethyl 6-*O*-benzyl-α-D-mannopyranosyl-(1→6)-[α-D-mannopyranosyl-(1→6)]_7_-D-mannopyranoside, and the glycosyl donor C_50_-polyprenol-phosphate-[^14^C]-mannose with membranes prepared from different *C. glutamicum* mutant strains, we have shown that both NCgl2100 and NCgl2097 encode for novel α(1→2)-mannopyranosyltransferases, which we have termed MptC and MptD respectively. Complementation studies and *in vitro* assays also identified Rv2181 as a homologue of Cg-MptC in *Mycobacterium tuberculosis*. Finally, we investigated the ability of LM and LAM from *C. glutamicum*, and *C. glutamicum*Δ*mptC* and *C. glutamicum*Δ*mptD* mutants, to activate Toll-like receptor 2. Overall, our study enhances our understanding of complex lipoglycan biosynthesis in *Corynebacterineae* and sheds further light on the structural and functional relationship of these classes of polysaccharides.

## Introduction

The *Corynebacterineae*, a sub-order of the Actinobacteria, represent an unusual group within Gram-positive bacteria, with a distinctive cell wall architecture. Prominent members are the human pathogens, *Corynebacterium diphtheriae*, *Mycobacterium tuberculosis* and *Mycobacterium leprae* (Bloom and Murray, 1992). Typically, the cell walls of *Corynebacterineae* contain mycolic acids (m), arabinogalactan (AG) and peptidoglycan (P), which are covalently linked to each other to form the mycolyl-arabinogalactan-peptidoglycan (mAGP) complex (Daffe *et al*., 1990; McNeil *et al*., 1990; 1991; Besra *et al*., 1995; 1997; Brennan, 2003; Dover *et al*., 2004). In addition, specialized glycophospholipids, phosphatidyl-*myo*-inositol mannosides (PIMs) and lipoglycans, lipomannan (LM) and lipoarabinomannan (LAM), are found in the outer leaflet of mAGP (Hill and Ballou, 1966; Brennan and Ballou, 1967; 1968; Brennan and Nikaido, 1995; Besra *et al*., 1997; Morita *et al*., 2004). The non-pathogenic bacillus, *Corynebacterium glutamicum,* also belongs to this class of bacteria, and is widely used in the industrial production of amino acids (Eggeling and Bott, 2005). Use of this easily cultivatable bacterium, together with the fact that the cell walls of *Corynebacterineae* share identical basic structures and building blocks, have contributed significantly to decipher the biosynthesis of their complex cell walls. For instance, in both *C. glutamicum* and *M. tuberculosis* the orthologous acyl-CoA carboxylase genes (Gande *et al*., 2007) together with Pks (Gande *et al*., 2004), are key to mycolic acid biosynthesis, and the glycosyltransferases AftA (Alderwick *et al*., 2006a), and AftB (Seidel *et al*., 2007a) introduce specific arabinofuranose (Ara*f*) residues into AG, and AftC in both AG and LAM of *Mycobacterium smegmatis* (Birch *et al*., 2008; 2010;). A speciation-specific difference is the presence of the glycosyltransferase RptA in *C. glutamicum* that introduces decorating rhamnose residues into AG (Birch *et al*., 2009), whereas a structurally very similar protein introduces galactosamine residues into AG of *M. tuberculosis* (Skovierova *et al*., 2010).

**Fig. 9:**
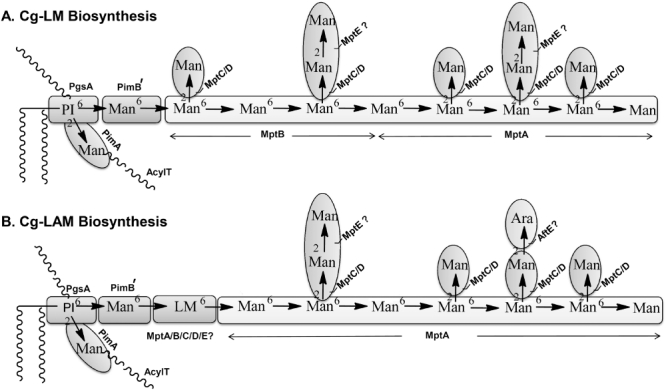
Biosynthetic pathway of LM (A) and LAM (B) biogenesis in *Corynebacterineae.* Cg-LM biosynthesis initially involves the formation of the α(1→6) mannan backbone by MptB, followed by α(1→2)-Man*p* branching by MptC (or MptD). This biosynthetic precursor would then be the substrate for further α(1→6)-Man*p* elongation by MptA, followed by α(1→2)-Man*p* branching by MptD (or MptC) to afford initially Cg-LM (A). Further extension by MptA along with additional GT-C glycosyltransferases further modify the α(1→6)-mannan backbone. These GT-Cs include MptC/D and MptE (formation of the dimannoside side-chain), and AftE/MptC/D and formation of Ara*f*-Man*p* side-chains (B).

A particular interesting group of cell wall components are the lipoglycans LM and LAM that are based on PIMs. They play poorly defined roles in *Corynebacterineae* but mycobacterial LAM has been implicated in many of the key aspects of the pathogenesis of tuberculosis and leprosy, such as induction of phagocytosis, phagosomal alteration and acquired T cell-mediated immunity (Briken *et al*., 2004). Mycobacterial LAM has attached to the PIM base, an elongated α(1→6) linear, α(1→2) branched mannan ‘core’, of approximately 30 mannopyranose (Man*p*) residues, and linked to its terminus a branched d-arabinan domain. In mycobacteria, this large arabinan domain consists of approximately 70 arabinofuranose (Ara*f*) residues and is assembled through several distinct motifs (Chatterjee *et al*., 1991; Alderwick *et al*., 2007; Birch *et al*., 2010), formed via specific α(1→5), α(1→3) and β(1→2) arabinofuranosyltransferases (Besra *et al*., 1997; Zhang *et al*., 2003; Alderwick *et al*., 2005; Birch *et al*., 2010), and can be capped to various degrees with either short α(1→2) mannopyranosyl chains, as is the case in *M. tuberculosis* (Chatterjee *et al*., 1992), or in *M. smegmatis* by inositol phosphate (Khoo *et al*., 1995), or completely devoid of caps as found in *Mycobacterium chelonae* (Guerardel *et al*., 2002). There is very limited structural information on LAM in other *Corynebacterineae*, except for *C. glutamicum* (Tatituri *et al*., 2007a,b;). In this organism the core α(1→6) linear α(1→2) branched LM (see Fig. 9), which is structurally akin to mycobacterial LM, is substituted by t-Ara*f* residues to form a modified LAM-like molecule (Tatituri *et al*., 2007a,b;).

The current model of lipoglycan biosynthesis supported by biochemical and genetic studies, follows a linear pathway from PI→PIM_2_→LM→LAM (Besra and Brennan, 1997; Besra *et al*., 1997). As with AG synthesis selected enzymatic and structural features are in part shared within the *Corynebacterineae*. Glycosylation of phosphatidyl-*myo*-inositol (PI) by different α-mannopyranosyltransferases and acylation by acyltransferase(s) results in the synthesis of mono- and di-acylated PIMs (Kordulakova *et al*., 2002; 2003; Kremer *et al*., 2002; Morita *et al*., 2006; Lea-Smith *et al*., 2008; Mishra *et al*., 2009). Previous studies have identified the enzymes involved in the early and late stages of LM and LAM biosynthesis in *Corynebacterineae*. For instance, NCgl1505/Rv1459c (MptB) and NCgl2093/Rv2174 (MptA), synthesize the proximal and distal α(1→6) mannan backbone in LM and LAM (Kaur *et al*., 2007; Mishra *et al*., 2007; 2008a;). However, the enzymatic steps involved in the biosynthesis of mannan branching of LM and LAM, still remain to be studied in detail in *Corynebacterineae* (Kaur *et al*., 2006; 2008; Sena *et al*., 2010).

In the current study we have examined two open reading frames from *C. glutamicum*, which encode for putative glycosyltransferase family C (GT-C) members (Liu and Mushegian, 2003). On the basis of mutant studies, chemical and enzymatic data we report that NCgl2100 (Cg-MptC) and its *M. tuberculosis* homologue Rv2181 function as α(1→2)-mannopyranosyltransferases, and NCgl2097 functions as a second α(1→2)-mannopyranosyltransferase (Cg-MptD), both of which are required for complete mannose-branching found in LM and LAM in *Corynebacterineae.*

## Results

### Genome locus and structural features of MptC and MptD

**Fig. 1:**
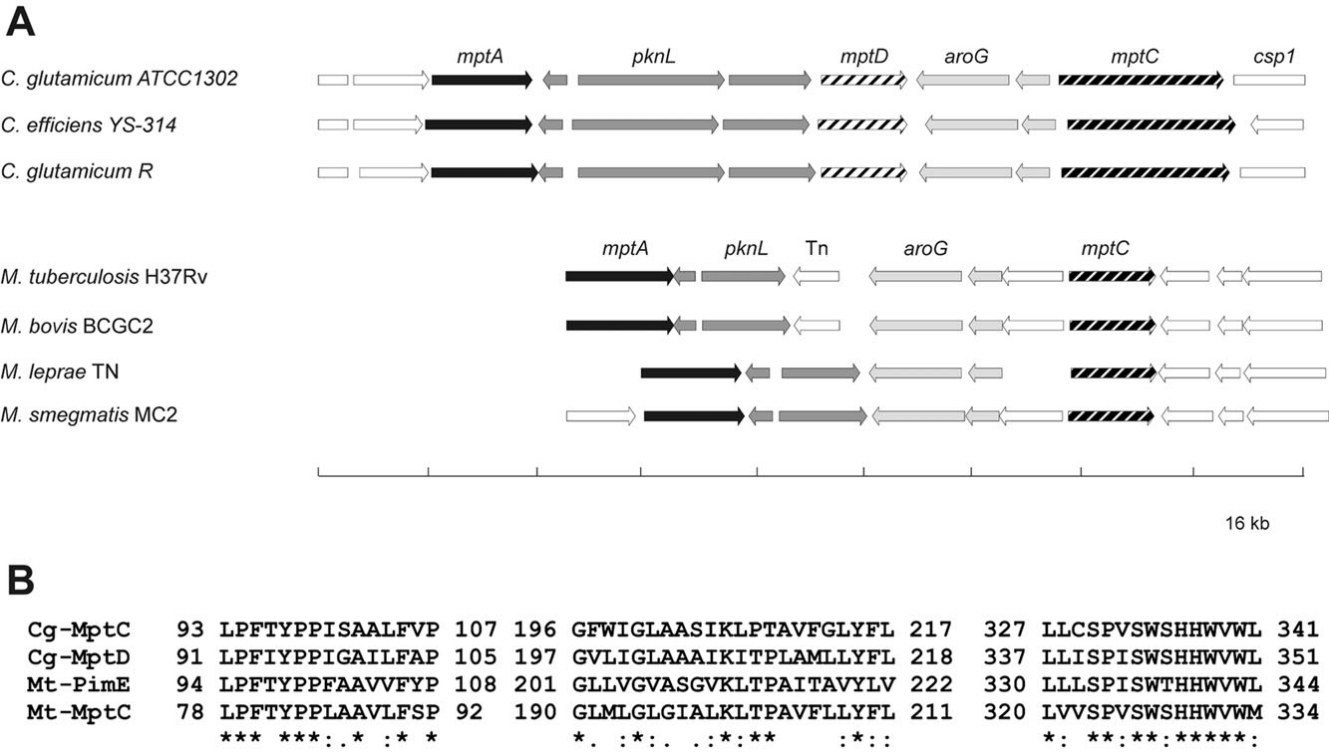
Comparative gene relatedness of α(1→2) and α(1→6) mannopyranosyltransferases. A. The genomic region in selected *Corynebacterianeae* containing the GT-C mannosyltransferases MptA, MptC and MptD. B. Partial sequence alignment of the strongly related α(1→2) mannosyltransferases Cg-MptC, Cg-MptD, Mt-PimE and Mt-MptC illustrating conserved residues.

We previously identified the α(1→6)-mannopyranosyltransferases, MptA and MptB, which catalyse the transfer of Man*p* residues from the glycosyl donor C_50_-polyprenol-phosphate mannose, to the distal and proximal ends of the mannan backbone of LM and LAM respectively (Mishra *et al*., 2007; 2008a;). In search of further GT-Cs, which synthesize the elaborated structures of LM and LAM, we interrogated the genome of *C. glutamicum* for additional GT-C family members (Liu and Mushegian, 2003). Located within a 16 kb genomic region containing *mptA*, which has been demonstrated to encode an α(1→2)-mannopyranosyltransferase in *M. tuberculosis* (Kaur *et al*., 2008), as does its orthologue *MSMEG_4247* in *M. smegmatis* (Kaur *et al*., 2006; Sena *et al*., 2010), are two additional GT-Cs (NCgl2100 [Cg-*mptC*], and NCgl2097 [Cg-*mptD*]) (Fig. 1A). In both *Corynebacterium* and *Mycobacterium* additional genes are retained at this locus: a serine/threonine protein kinase (*pknL*), hypothetical proteins (NCgl2099, Rv2179c; NCgl2094, Rv2175c) and a 3-deoxy-7-phosphoheptulonate synthase (*aroG*) (Fig. 1A).

The *Cg-mptC* encoded protein is a long hydrophobic polytopic membrane protein of 812 amino acid (aa) residues. The first half of the protein (1–417 aa), shares 37% identity (55% similarity) with Mt-MptC and 38% identity (57% similarity) to Cg-MptD, whereas the second half (417–812 aa), has no counterpart in various *Mycobacterium* species. The protein appears to be a fusion of two membrane proteins, functional as an α(1→2)-mannopyranosyltransferase as described below, and termed in this study Cg-MptC. Cg-MptD is a hydrophobic polytopic membrane protein of 436 amino acid residues with 11 transmembrane helixes (TMH). After TMH-1 and TMH-7, larger loop regions are present probably localized in the periplasm. Cg-MptC, Cg-MptD and Mt-MptC, together with Mt-PimE, an α(1→2)-mannopyranosyltransferase demonstrated to be involved in PIM_6_ synthesis (Morita *et al*., 2006), share a similar size, degree of hydrophobicity and regions of high identity (Fig. 1B), suggesting closely related functions.

### Construction of deletion mutants and growth phenotype

The construct pK19mobsacBΔCg-*mptC* was made containing 12 nucleotides (nt) of the 3′ end of *NCgl2100* together with the genomic upstream sequences, and 36 nt of the 5′ end together with genomic downstream sequences (Table S1). This non-replicative vector was used to transform *C. glutamicum* to kanamycin-resistance (Kan^r^) indicating chromosomal integration. Sucrose-resistant (Suc^r^) clones were selected in a second round of positive selection, indicating loss of the vector-encoded *sacB* function (Schäfer *et al*., 1994). From 12 Kan^s^ and Suc^r^ clones analysed via PCR, seven had lost *NCgl2100*, whereas in the remaining five the genomic wild type situation was restored. One clone with deleted *NCgl2100* was selected and will be referred to as *C. glutamicum*Δ*mptC* (Fig. S1) In an analogous manner, pK19mobsacBΔCg-*mptD* was constructed and used to select from the wild type of *C. glutamicum* by double cross-over events as described above for deletion of *Cg-mptD*, yielding *C. glutamicum*Δ*mptD* (Fig. S1). Starting from *C. glutamicum*Δ*mptC* and using pK19mobsacBΔCg-*mptD* the double deletion mutant *C. glutamicum*Δ*mptC*Δ*mptD* was also constructed (Fig. S1).

The mutants were inoculated into liquid BHI and CGXII medium and their growth patterns studied. There was no statistical difference observed in three independent cultivations for each of the strains. The growth rates in CGXII were 0.39 ± 0.02 h^−1^ for wild-type *C. glutamicum*, *C. glutamicum*Δ*mptC* and *C. glutamicum*Δ*mptD*, and 0.37 ± 0.02 h^−1^ for *C. glutamicum*Δ*mptC*Δ*mptD* (data not shown). The growth rates on BHI medium were 0.66 ± 0.07 h^−1^ for all three strains and illustrate that the genes are not essential for growth as observed for other GT-Cs involved in mAGP synthesis like, Cg-*emb*, Cg-*aftA* and Cg-*aftB* (Alderwick *et al*., 2005; 2006a; Seidel *et al*., 2007a).

### Purification and general characteristic features of lipoglycans from *C. glutamicumΔmptC* and *C. glutamicum*Δ*mptD*

**Fig. 2:**
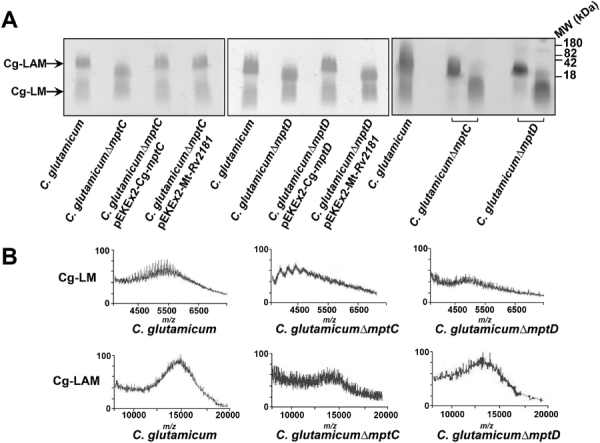
Lipoglycan profiles of wild-type and mutant strains of *C. glutamicum*. A. The left-hand and middle panels illustrate lipoglycans extracted from wild-type, mutant and complemented strains analysed using SDS-PAGE and visualized using a Pro-Q emerald glycoprotein stain (Invitrogen). The right-hand panel illustrates purified Cg-LAM and Cg-LM from *C. glutamicumΔmptC* and *C. glutamicumΔmptD*, respectively, in comparison with wild-type *C. glutamicum* lipoglycans. Lipoglycan profiles are represented with standard molecular weight markers of glycoproteins of 180, 82, 42 and 18 kDa. B. MALDI-TOF-MS spectra of Cg-LM and Cg-LAM from different strains of *C. glutamicum*. MALDI-TOF-MS spectra were acquired in the linear negative mode with delayed extraction using 2,5-dihydrobenzoic acid as a matrix.

Lipoglycans from wild-type *C. glutamicum*, *C. glutamicumΔmptC* and *C. glutamicumΔmptD* with complemented strains *C. glutamicumΔmptC* pEKEx2-Cg-*mptC*, *C. glutamicumΔmptC* pEKEx2-*Rv2181*, *C. glutamicumΔmptD* pEKEx2-Cg-*mptD* and *C. glutamicumΔmptD* pEKEx2-*Rv2181*, were extracted from delipidated cells by ethanol/water extraction followed by hot-phenol water treatment and enzymatic degradation. The crude lipoglycan extract was subjected to hydrophobic interaction and gel permeation chromatography allowing the separation of Cg-LM (Cg-LM-A and Cg-LM-B) and Cg-LAM (Tatituri *et al*., 2007b; Lea-Smith *et al*., 2008; Mishra *et al*., 2008b). Because the mannan backbone structures of Cg-LM-A and Cg-LM-B are similar as revealed through previous matrix*-*assisted laser desorption/ionization time-of-flight mass spectrometry (MALDI-TOF-MS), and gas chromatography-mass spectrometry (GC-MS) analyses of partially methylated alditol acetates (Mishra *et al*., 2008b), subsequent analyses focused on the Cg-LM as a mixture. The extracted lipoglycans were examined for their size and mobility on 15% SDS-PAGE (Fig. 2A). The crude extracts from wild-type *C. glutamicum* showed the presence of Cg-LM and Cg-LAM (Tatituri *et al*., 2007a; Lea-Smith *et al*., 2008; Mishra *et al*., 2008b) (Fig. 2A), while the crude lipoglycans from mutant strains, *C. glutamicumΔmptC* and *C. glutamicumΔmptD*, showed subtle differences in their migration on SDS-PAGE (Fig. 2A). Purified Cg-LAM from *C. glutamicumΔmptC* and *C. glutamicumΔmptD* (Fig. 2A) migrated faster on SDS-PAGE in comparison with those from wild-type *C. glutamicum*, indicating that these lipoglycans were smaller in size with low molecular weights. In addition, fractionation using gel permeation chromatography also revealed a degree of heterogeneity in Cg-LM and Cg-LAM, as evident when fractions were pooled. Complementation of *C. glutamicumΔmptC* and *C. glutamicumΔmptD* by pEKEx2-Cg-*mptC* and pEKEx2-Cg-*mptD*, respectively, restored the wild-type phenotype, as illustrated by the slower and co-migration on SDS-PAGE in comparison with wild-type *C. glutamicum* (Fig. 2A). In contrast, cross-complementation experiments, *C. glutamicumΔmptC* by pEKEx2-Cg-*mptD*, and *C. glutamicumΔmptD* by pEKEx2-Cg-*mptC*, failed to restore a wild-type phenotype and reversion to a slower migration of products on SDS-PAGE (Fig. S2), as observed in our previous experiments described above (Fig. 2A).

The molecular weights of the lipoglycans were investigated by MALDI-TOF-MS. The Cg-LAM from wild-type *C. glutamicum* exhibited a broad unresolved peak centred at m/z 15 000 (approximately 85 Man*p*/Ara*f* glycosyl residues), and Cg-LM at m/z 5500 (approximately 25 Man*p* residues), indicating a molecular weight of approximately 15 and 5.5 kDa for the major molecular species of these lipoglycans (Fig. 2B). Interestingly, Cg-LAM from *C. glutamicumΔmptC* (Fig. 2A) peaked at m/z 13 800 (approximately 78 Man*p*/Ara*f* glycosyl residues), indicating a decrease of around 1.2 kDa for the Cg-LAM isolated from *C. glutamicumΔmptC* as compared with wild-type Cg-LAM (Fig. 2B). In addition, Cg-LM from *C. glutamicumΔmptC* (Fig. 2A) exhibited a broad unresolved peak centred at m/z 4400 (approximately 20 Man*p* glycosyl residues), indicating a molecular weight of approximately 4.4 kDa, a decrease of 1.1 kDa in comparison with wild-type *C. glutamicum* (Fig. 2B). Similarly, the lipoglycans, Cg-LM and Cg-LAM from *C. glutamicumΔmptD* (Fig. 2A) are smaller and centred around m/z 13 000 (approximately 73 Man*p*/Ara*f* glycosyl residues), and 4700 (approximately 20 Man*p* glycosyl residues) respectively (Fig. 2B). The differences in size of Cg-LAM and Cg-LM from the mutant strains, suggest that there is a difference in a common component of both mutant strains, which also reflects the earlier observation on SDS-PAGE (Fig. 2A).

### Glycosyl composition of purified lipoglycans from *C. glutamicum*Δ*mptC* and *C. glutamicum*Δ*mptD*

Purified lipoglycans from wild-type *C. glutamicum*, *C. glutamicumΔmptC* and *C. glutamicumΔmptD* were analysed as alditol acetates and their glycosyl composition was determined by gas chromatography (GC) (Fig. S3). GC analysis of alditol acetates prepared from wild-type Cg-LAM revealed a molar ratio of Ara*f* : Man*p* of 0.47:1.0. The mutant Cg-LAM from *C. glutamicumΔmptC* and *C. glutamicumΔmptD* yielded a significant reduction in Man*p* content concomitant with a relative increase in the amount of Ara*f*. The *C. glutamicumΔmptC* yielded a mutant Cg-LAM with an Ara*f* : Man*p* ratio of 0.59:1.0, while Cg-LAM from *C. glutamicumΔmptD*, yielded an Ara*f* : Man*p* ratio of 0.7:1.0. The data suggest that both Cg-MptC and Cg-MptD are involved in the synthesis of the mannan portion of Cg-LAM together with Cg-MptA and Cg-MptB (Mishra *et al*., 2007; 2008a;).

### Glycosyl linkage of purified lipoglycans from *C. glutamicumΔmptC* and *C. glutamicumΔmptD*

**Fig. 3:**
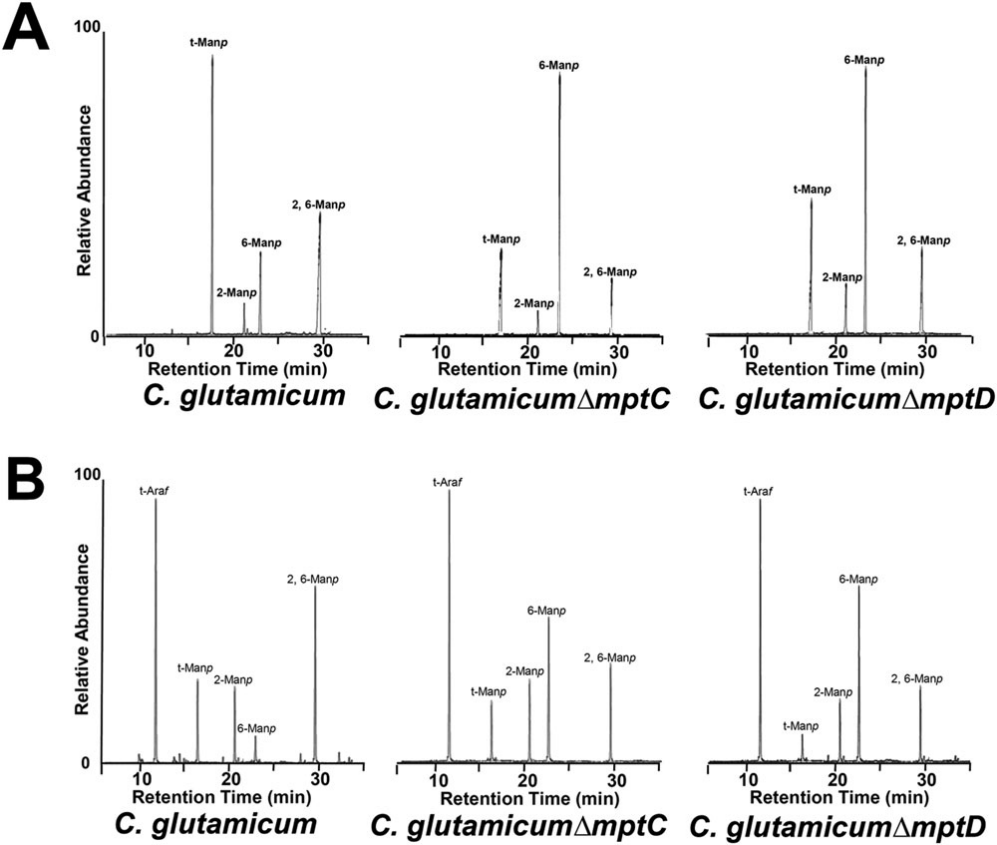
Glycosyl linkage analysis of Cg-LM and Cg-LAM from different strains of *C. glutamicum*. A. Cg-LM per-*O*-methylated samples were hydrolysed using 2 M TFA, reduced and per-*O*-acetylated. The resulting partially per-*O*-methylated, per-*O*-acetylated alditol acetates were analysed using GC-MS. B. Cg-LAM per-*O*-methylated samples were hydrolysed using 2 M TFA, reduced and per-*O*-acetylated. The resulting partially per-*O*-methylated, per-*O*-acetylated alditol acetates were analysed using GC-MS. The partially per-*O*-methylated, per-*O*-acetylated alditol acetates were identified based on their characteristic alditol cleavage acetal profiles: t-Ara*f* (m/z 118, 161), t-Man*p* (m/z 118, 102, 145, 161, 162, 205), 2-Man*p* (m/z 129, 130, 161, 190), 6-Man*p* (m/z 118, 129, 162, 189, 233) and 2,6-Man*p* (m/z 129, 130, 189, 190).

The glycosyl linkages present in the lipoglycans from wild-type and mutant strains of *C. glutamicum* were analysed by GC-MS of per-*O*-methylated alditol acetate derivatives prepared from purified lipoglycans. Cg-LM from *C. glutamicum* indicated a normal profile of glycosidic linkages corresponding to t-Man*p* (49%), 6-Man*p* (15%), 2-Man*p* (6%) and 2,6-Man*p* (30%) (Fig. 3A). However, the relative ratios of different linkages in Cg-LM from *C. glutamicumΔmptC*[t-Man*p* (23%), 6-Man*p* (58%), 2-Man*p* (6%) and 2,6-Man*p* (13%)] and *C. glutamicumΔmptD*[(t-Man*p* (25%), 6-Man*p* (45%), 2-Man*p* (8%) and 2,6-Man*p* (22%)] indicated subtle changes in the mannan domain in Cg-LM from these strains consistent with the earlier MALDI-TOF-MS data indicating a reduction in size (Fig. 2B). The relative abundance of t-Man*p* and 2,6-Man*p* were reduced with a concomitant increase in the abundance of 6-Man*p* in Cg-LM from these strains (Fig. 3A). Similarly, per-*O*-methylated alditol acetate derivatives of Cg-LAM from these strains showed a net reduction in relative abundance of t-Man*p* and 2,6-Man*p* and increase in 6-Man*p* (Fig. 3B). For instance, Cg-LAM from *C. glutamicum* indicated a normal profile of glycosidic linkages corresponding to t-Ara*f* (40%), t-Man*p* (14%), 6-Man*p* (6%), 2-Man*p* (11%) and 2,6-Man*p* (29%) (Fig. 3A). Whereas the relative ratios of different linkages in Cg-LAM from *C. glutamicumΔmptC* were t-Ara*f* (40%), t-Man*p* (6%), 6-Man*p* (30%), 2-Man*p* (11%) and 2,6-Man*p* (13%) and *C. glutamicumΔmptD* were t-Ara*f* (40%), t-Man*p* (8%), 6-Man*p* (26%), 2-Man*p* (12%) and 2,6-Man*p* (14%). The complementation of *C. glutamicumΔmptC* and *C. glutamicumΔmptD* with pEKEx2-Cg-*mptC* and pEKEx2-Cg-*mptD,* respectively, restored the wild-type phenotype in these strains (data not shown). Altogether, the data together suggest that Cg-LM and Cg-LAM from *C. glutamicumΔmptC* and *C. glutamicumΔmptD* have reduced 2,6-Man*p* branching residues indicating that MptC and MptD are involved in the synthesis of α(1→2)-Man*p* residues in Cg-LM and Cg-LAM in *C. glutamicum*.

### Characterization of Cg-LAM from *C. glutamicumΔmptCΔmptD*

**Fig. 4:**
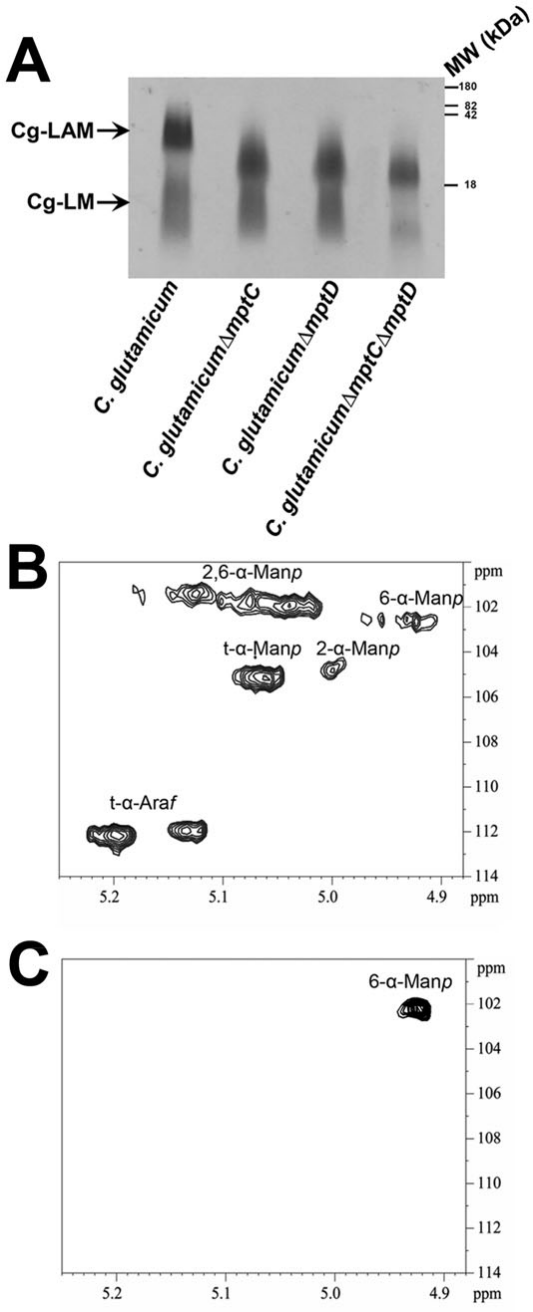
Lipoglycan analysis of *C. glutamicumΔmptCΔmptD*. A. Lipoglycans were extracted from wild-type and mutant strains and were analysed using SDS-PAGE and visualized using a Pro-Q emerald glycoprotein stain (Invitrogen). Lipoglycan profiles are represented with standard molecular weight markers of glycoproteins of 180, 82, 42 and 18 kDa. B. 2D ^1^H-^13^C-NMR HMQC characterization of Cg-LAM from *C. glutamicum* as described in Tatituri *et al*. (2007b). C. 2D ^1^H-^13^C-NMR HMQC characterization of Cg-LAM from *C. glutamicumΔmptCΔmptD*. NMR spectra of Cg-LAMs were recorded in D_2_O at 313 K.

Because the data in Fig. 3A,B suggest that deletion of *mptC* or *mptD* results in only partial removal of 2,6-Man*p* branching residues, we constructed a *C. glutamicumΔmptCΔmptD* double mutant strain (as described earlier). The extracted lipoglycans were examined for their size and mobility on 15% SDS-PAGE (Fig. 4A). The crude lipoglycan extracts of *C. glutamicumΔmptCΔmptD* in comparison with wild-type *C. glutamicum*, *C. glutamicumΔmptC* and *C. glutamicumΔmptD*, migrated faster on SDS-PAGE, indicating that these lipoglycans were smaller in size (Fig. 4A). The purified Cg-LAM from *C. glutamicumΔmptCΔmptD* was analysed by ^1^H-^13^C-NMR HMQC (Fig. 4C) and compared with our previously reported ^1^H-^13^C-NMR HMQC (Fig. 4B) assignment of anomeric resonances of Cg-LAM from *C. glutamicum* (Tatituri *et al*., 2007b). Briefly, correlations at δ_H1C1_ 5.20/112.2 and 5.13/112.0 were attributed to t-α-Ara*f* units; 5.06/105.2 to t-α-Man*p* units; 5.12/101.4, 5.07/10.1.7, and 5.04/101.9 to 2,6-α-Man*p* units; 4.93/102.6 to 6-α-Man*p* units; and 5.00/104.9 to 2-α-Man*p* units, which were consistent with our above glycosyl linkage data for Cg-LAM from *C. glutamicum* (Fig. 2B). In contrast, the ^1^H-^13^C-NMR HMQC (Fig. 4C) of Cg-LAM from *C. glutamicumΔmptCΔmptD* was dominated by a single anomeric resonance at δ_H1C1_ 4.92/102.5 and assigned to 6-α-Man*p* units, consistent with a complete removal of 2,6-α-Man*p* branching residues. GC-MS of per-*O*-methylated alditol acetate derivatives from Cg-LAM purified from *C. glutamicumΔmptCΔmptD* confirmed these results and the identification of 2,3,4-tri-*O*-CH_3_-1,5,6-tri-*O*-COCH_3_-mannitol with the characteristic fragment alditol cleavage ions m/z 102, 118, 129, 162, 189 and 233 (Fig. S4).

### *In vitro*α(1→2)-mannopyranosyltransferase assay utilizing a novel blocked nonasaccharide acceptor

**Fig. 5:**
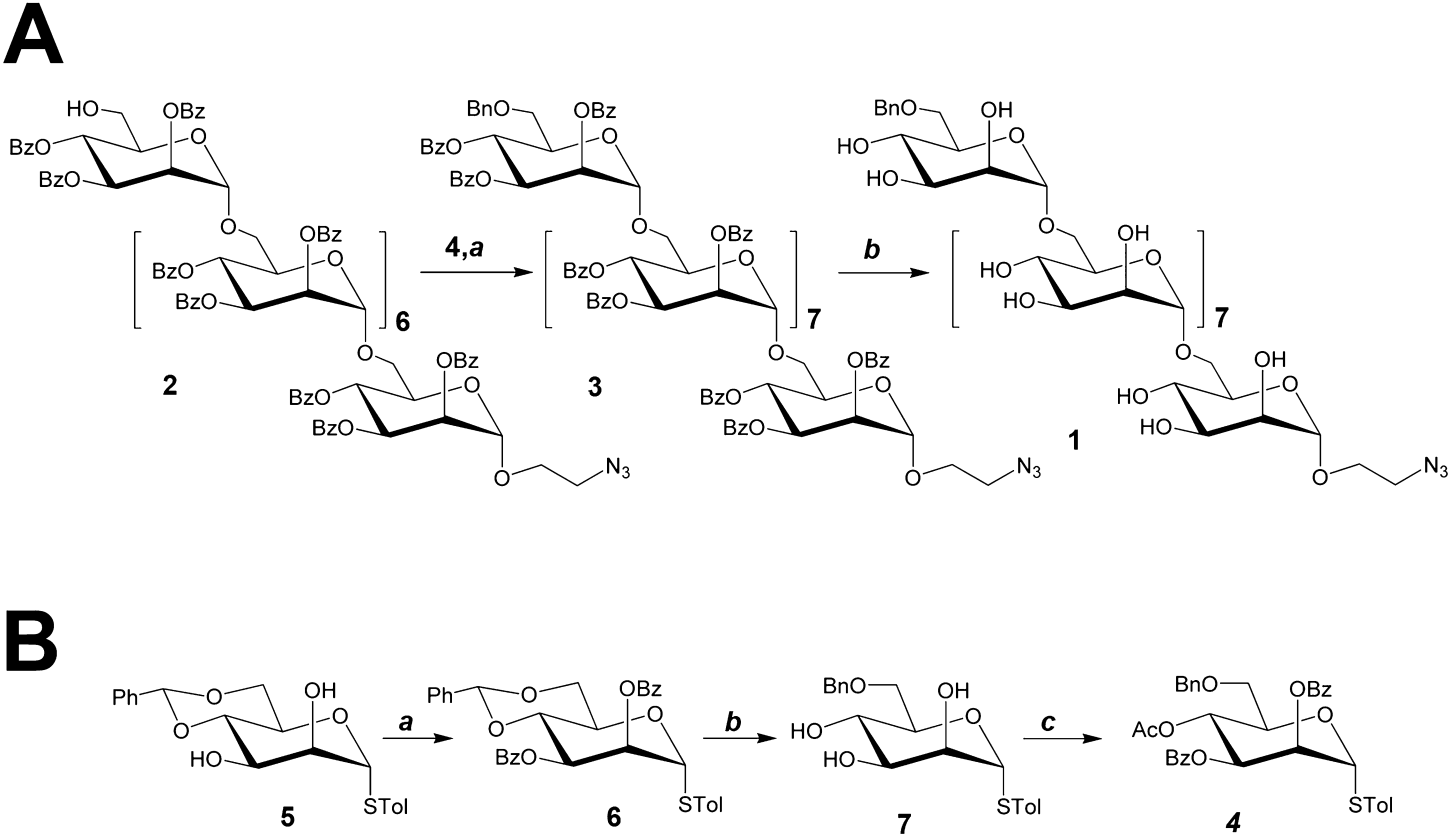
Chemical synthesis of the nonasaccharide acceptor azidoethyl 6-*O*-benzyl-α-**D**-mannopyranosyl-(1→6)-[α-**D**-mannopyranosyl-(1→6)]_7_-**D**-mannopyranoside (1) and key intermediate 4. Reagents and conditions. A. (a) NIS, triflic acid, CH_2_Cl_2_, −20°C–0°C, 1 h, 82%; (b) Sodium methoxide, CHCl_3_-CH_3_OH (1:2), overnight, 35%; B. (a) BzCl, pyridine, room temperature, 4 h, 94%; (b) Et_3_SiH, triflic acid, CH_2_Cl_2_, −78°C, 1 h, 80%; (c) acetic anydride, pyridine, room temperature, 5 h, 94%.

For characterization of α(1→2)-mannopyranosyltransferase activities of Cg-MptC and Cg-MptD a nonasaccharide acceptor, azidoethyl 6-*O*-benzyl-α-**D**-mannopyranosyl-(1→6)-[α-**D**-mannopyranosyl-(1→6)]_7_-**D**-mannopyranoside, Acc-Man_9_ (Fig. 5A, compound **1**) was chemically synthesized (Fig. 5A,B). The synthesis of the target nonamannopyranoside **1** was planned starting from the previously synthesized **2** (Aqueel *et al*., 2008) via synthesis of oligosaccharide **3** (Fig. 5A). For the synthesis of **3**, donor thioglycoside **4** was synthesized starting from the reported 5,6-*O*-benzylidene protected thioglycoside **5** (Cumpstey *et al*., 2004) (Fig. 5B). In a straight forward reaction sequence, thioglycoside **5** was treated with benzoyl chloride in pyridine to access the thioglycoside **6**, which on regioselective ring opening with Et_3_SiH gave 6-OBn thioglycoside **7**. The ^1^H NMR and ^13^C NMR spectral studies confirmed the structure of **7**. An acetate protecting group was installed at the 4-OH position in thioglycoside **7** by reacting with acetic anhydride in pyridine resulting in the thioglycoside donor **4**. An acetate group was specifically chosen to easily characterize the reaction product during coupling of **4** and **2** by NMR. The donor thioglycoside **4** and **2** were coupled in the presence of NIS and triflic acid at −20°C, resulting in nonasaccharide **3** after chromatographic purification. The observed singlet signal in the ^1^H NMR spectrum of **3** at δ 1.88 ppm was assigned to the three protons of the acetyl protecting group, and the resonances at δ 4.48 and 4.28 ppm (doublets, *J* = 11.9 Hz), for one proton each to the benzyl protecting group, supporting the structure of **3**. The ^13^C NMR spectrum further confirmed the structure of **3,** with the presence of signals at 73.45 and 20.81 ppm, which were assigned to the CH_2_ of the benzyl and CH_3_ of the acetyl carbons respectively. The ester protecting groups in oligosaccharide **3** were removed in an overnight reaction using sodium methoxide in CH_3_OH. The reaction was neutralized using H^+^ Amberlite resin and the reaction product purified using Bio-Beads SM-4 using a CH_3_OH-H_2_O mobile phase and 0–60% gradient resulting in the desired nonomannoside **1**. The ^1^H NMR spectrum of **1** showed the presence of benzyl signals at δ 4.64 and 4.61 ppm (doublet, *J* = 11.7 Hz). In the ^13^C NMR spectrum, the benzyl carbon was observed at δ 73.32 ppm and CH_2_N_3_ at δ 51.79 ppm. Finally, the structure of **1** was further supported by HR-MS, which showed a [M + Na]^+^ peak at 1685.55.

**Fig. 6:**
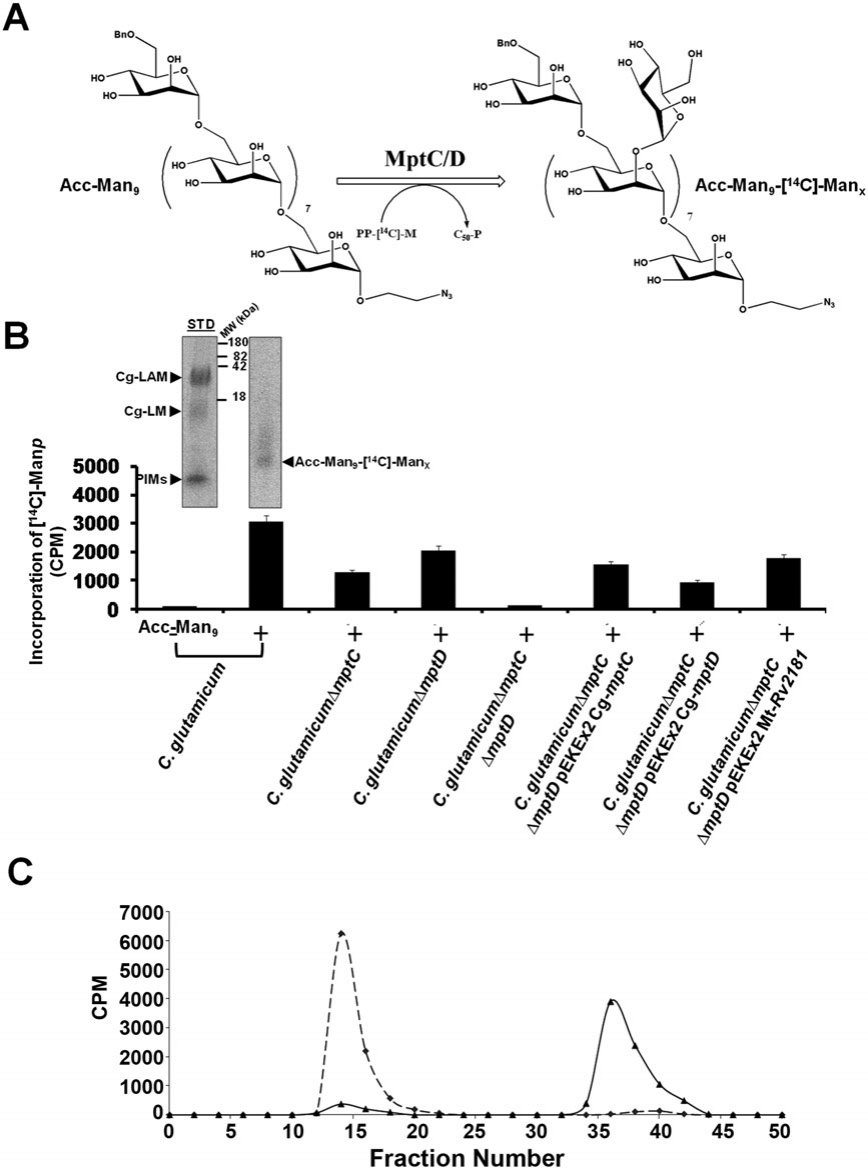
*In vitro*α(1→2)-mannopyranosyltransferase assay utilizing a nonasaccharide acceptor. A. Reaction scheme illustrating *in vitro* assays performed with the chemically synthesized nonasaccharide acceptor, azidoethyl 6-*O*-benzyl-α-**D**-mannopyranosyl-(1→6)-[α-**D**-mannopyranosyl-(1→6)]_7_-**D**-mannopyranoside, Acc-Man_9,_ and either MptC or MptD, resulting in the synthesis of Acc-Man_9_-[^14^C]-Man_x_. B. The reactions were set up with and without Acc-Man_9_ (+ or −), PP-[^14^C]-M, and membrane preparations from different strains of *C. glutamicum* as described in the *Experimental procedures*. C. The Acc-Man_9_-[^14^C]-Man_x_ product from the use of *C. glutamicum* membranes was dried and subsequently incubated with an α(1→2)-mannosidase. The reaction mixture was then fractionated on a Bio-Gel P-2 gel filtration column. The control Acc-Man_9_-[^14^C]-Man_x_ (dotted line) product before α(1→2)-**D**-mannosidase treatment eluted from the Bio-Gel P-2 column at fractions 11–13, and the digested product, released [^14^C]-Man (solid line), was retained and eluted in later fractions 33–39, co-eluting with a [^14^C]-mannose standard (Cooper *et al*., 2002).

The structural features of the acceptor allowed its specific isolation and purification of *in vitro* reaction products synthesized by distinct α(1→2)-mannopyranosyltransferases using a strong anion exchange cartridge, thus removing from the *in vitro* assay unused C_50_-polyprenol-phospho-[^14^C]-mannose (PP-[^14^C]-M) and other *in vitro* synthesized products, such as higher [^14^C]-PIMs, [^14^C]-LM and [^14^C]-LAM. In addition, the non-reducing sugar of **1** (which we have abbreviated as Acc-Man_9_) is blocked at the *O*-6 position by a benzyl ether, thus preventing further elongation by endogenous activities through MptA and MptB via α(1→6)-mannopyranosyltransferases. Overall, the utilization of Acc-Man_9_ and the *in vitro* assay provides a novel route to evaluate distinct α(1→2)-mannopyranosyltransferases in *Corynebacterineae* (Fig. 6A). The *in vitro* transfer of [^14^C]-Man*p* from PP-[^14^C]-M onto the Acc-Man_9_ acceptor was examined using membrane preparations from wild-type and mutant strains of *C. glutamicum* (Fig. 6B). In this improved assay, membrane preparations from wild-type *C. glutamicum* transferred 3072 ± 132 cpm of [^14^C]-Man*p* from PP-[^14^C]M onto the synthetic Acc-Man_9_ via an α(1→2)-mannopyranosyltransferase (Fig. 6B and inset). Assays repeated in the absence of Acc-Man_9_ afforded background activity 102 ± 12 cpm, demonstrating the efficiency of the assay (Fig. 6B). Membrane preparations from both *C. glutamicum*Δ*mptC* and *C. glutamicum*Δ*mptD* used in above assays afforded α(1→2)-mannopyranosyltransferase activities, but in each case the relative level of activity in comparison with wild-type *C. glutamicum* membranes was reduced (Fig. 6B).

The analysis of [^14^C] reaction products was simplified further by utilizing membrane preparations from the branching deficient double mutant, *C. glutamicum*Δ*mptC*Δ*mptD*, lacking Cg-MptC and Cg-MptD. The *C. glutamicum*Δ*mptC*Δ*mptD* mutant possessed background (149 ± 15 cpm) incorporation of [^14^C]-Man*p* (Fig. 6B), demonstrating the complete absence of α(1→2)-mannopyranosyltransferase activity in this double mutant. Therefore, the double mutant, *C. glutamicum*Δ*mptC*Δ*mptD* was used to analyse the enzymatic activities of Cg-MptC and Cg-MptD. Cg-*mptC* and Cg-*mptD* were cloned into pEKEx2 and introduced into *C. glutamicum*Δ*mptC*Δ*mptD* resulting in *C. glutamicum*Δ*mptC*Δ*mptD* pEKEx2-Cg-*mptC* and *C. glutamicum*Δ*mptC*Δ*mptD* pEKEx2-Cg-*mptD*. *C. glutamicum*Δ*mptC*Δ*mptD* pEKEx2-Cg-*mptC* resulted in 1570 ± 83 cpm of Acc-Man_9_-[^14^C]-Man_x_, while *C. glutamicum*Δ*mptC*Δ*mptD* pEKEx2-Cg-*mptD* resulted in 953 ± 24 cpm of Acc-Man_9_-[^14^C]-Man_x_.

### Acc-Man_9_-[^14^C]-Man_x_ selective cleavage by an α(1→2)-D-mannosidase

The extracted Acc-Man_9_-[^14^C]-Man_x_ reaction product from the above assays performed with *C. glutamicum* membranes (Fig. 6B) were dried and subsequently treated with a previously characterized α(1→2)-**D**-mannosidase from *Trichoderma reesei* (Maras *et al*., 2000) as described (Ichishima *et al*., 1981; Yokoyama and Ballou, 1989) to determine the α(1→2) addition of Man*p*. The digested mixtures were fractionated on a gel filtration column. The control Acc-Man_9_-[^14^C]-Man_x_ reaction product, before α(1→2)-**D**-mannosidase treatment eluted from the Bio-Gel P-2 column at fractions 13–16, and this completely enzymatically digested product (measured through the release of [^14^C]-Man*p*), was retained and eluted in later fractions 35–42 with a [^14^C]-mannose standard (Fig. 6C; Cooper *et al*., 2002). The results demonstrate that the addition of [^14^C]-Man*p* to Acc-Man_9_ is via an α(1→2)-mannopyranosyltransferase, and combined with the data from Fig. 6B and the use of the *C. glutamicum*Δ*mptC*Δ*mptD* mutant strain, has shown that MptC and MptD are solely responsible for this α(1→2)-mannopyranosyltransferase activity.

### Functional identification of the mycobacterial homologue of Cg-MptC

**Fig. 7:**
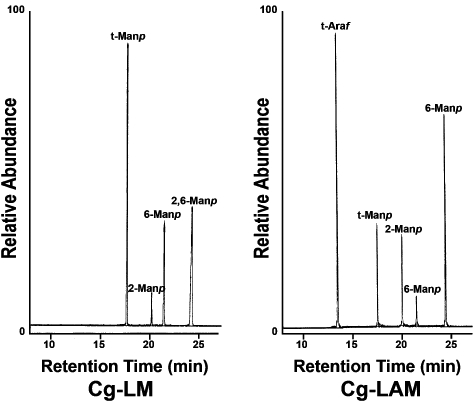
Characterization of the mycobacterial homologue Rv2181 of Cg-MptC. Lipoglycans were extracted from *C. glutamicumΔmptC* pEKEx2-Mt-*Rv2181*, and the resulting Cg-LM and Cg-LAM products purified*,* per-*O*-methylated, and hydrolysed using 2 M TFA, reduced and per-*O*-acetylated. The resulting partially per-*O*-methylated, per-*O*-acetylated alditol acetates were analysed by GC-MS and products identified based on their characteristic alditol cleavage acetal profiles: t-Ara*f* (m/z 118, 161), t-Man*p* (m/z 118, 102, 145, 161, 162, 205), 2-Man*p* (m/z 129, 130, 161, 190), 6-Man*p* (m/z 118, 129, 162, 189, 233) and 2,6-Man*p* (m/z 129, 130, 189, 190).

As described above (Fig. 1), Cg-MptC showed highest similarity of 55% to Rv2181 of *M. tuberculosis*, while Cg-MptD showed weaker, but significant similarity of 47.9% to Rv2181. To assay for a complementary function of Rv2181 in *C. glutamicum*, plasmid pEKEx2-Mt-*Rv2181* was constructed. Upon introduction into *C. glutamicumΔmptC*, the wild-type phenotype of *C. glutamicum* was restored (Fig. 2A) illustrating that Mt-MptC is functional and utilizes the corynebacterial substrates. In addition, Rv2181 was very specific for *C. glutamicumΔmptC* as complementation of the mutant phenotype in *C. glutamicumΔmptD* was not obtained (Fig. 2A). The complementation of *C. glutamicumΔmptC* with pEKEx2-Mt-*Rv2181* also restored the wild-type glycosyl linkage phenotype in this strain (Fig. 7, and see Fig. 3A and B). Furthermore, use of membrane preparations from *C. glutamicum*Δ*mptC*Δ*mptD* pEKEx2-Mt-*Rv2181* and the synthetic Acc-Man_9_ (Fig. 6A) resulted in the incorporation of 1811 ± 87 cpm of [^14^C]-Man*p* into the [^14^C]-mannan product (Fig. 6B). Altogether, these data strongly suggest that *Rv2181* is a homologue of Cg-*mptC* and both encode for α(1→2)-mannopyranosyltransferase activities, involved in the synthesis of the α(1→2)-Man*p* branches of LM and LAM in *Corynebacterineae*.

### Activation of Toll-like receptor 2 (TLR2) by wild-type and mutant Cg-LM and LAM

**Fig. 8:**
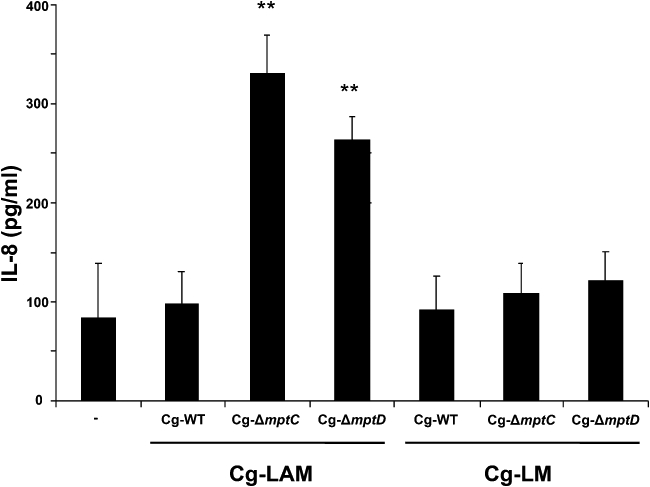
TLR-2 stimulation by wild-type and mutant Cg-LM and Cg-LAM. HEK293 cells transfected with TLR2 were stimulated with 50 µg ml^−1^ of wild-type (WT) or mutant (Δ*mptC*/Δ*mptD*) Cg-LAM or Cg-LM. Unstimulated cells (−) served as a control. After 24 h of stimulation (37°C), supernatants were harvested and analysed for IL-8 content using ELISA. The results represent the mean IL-8 production ± SD from three independent experiments (each experiment was performed in triplicate). Double asterisks indicate highly significant (*P* < 0.001) differences as compared to the unstimulated control cells.

Both LM and LAM are bioactive molecules thought to be involved in modulating the host immune response (Briken *et al*., 2004). Bacterial lipoglycans are recognized by a variety of host immune receptors including TLR2. In fact, mycobacterial LMs are well known to be potent activators of this latter receptor hence providing them strong pro-inflammatory activity (Vignal *et al*., 2003; Quesniaux *et al*., 2004; Gilleron *et al*., 2006). On the other hand, LAMs are only poorly active; a feature attributed to the presence of the arabinan domain that masks the ‘bioactive’ mannan core (Vignal *et al*., 2003; Nigou *et al*., 2008; Birch *et al*., 2010). To investigate the ability of Cg-LM and Cg-LAM to activate TLR2 to assess possible consequences of Cg-*mptC* and Cg-*mptD* inactivation in this process, purified LM and LAM from *C. glutamicum*Δ*mptC* and *C. glutamicum*Δ*mptD,* were used to stimulate HEK293 cells expressing TLR2. To prevent interference of co-purified lipopeptides, the glycolipids were first pretreated with hydrogen peroxide (Morr *et al*., 2002; Geurtsen *et al*., 2009; Birch *et al*., 2010). As shown in Fig. 8, wild-type Cg-LAM was unable to activate TLR2. This result is consistent with an earlier study in which it was shown that LAMs substituted with single arabinosyl residues were unable to induce the production of tumour necrosis factor-α in human THP-1 monocytes (Nigou *et al*., 2008). Interestingly, LAMs isolated from the *C. glutamicum*Δ*mptC* and *C. glutamicum*Δ*mptD* mutant strains demonstrated an increased activity as evidenced by the enhanced TLR2-dependent IL-8 production by HEK293 cells (Fig. 8). This suggests that partial removal of α(1→2)-Man*p* units, increases the ability of Cg-LAM to activate TLR2. In contrast to mycobacterial LMs, wild-type Cg-LM did not show any activity towards TLR2. Furthermore, mutation of Cg-*mptC* and Cg-*mptD* did not alter this behaviour and the mutant LMs remained inactive (Fig. 8). These results demonstrate that wild-type Cg-LAM and Cg-LM, are both TLR2 inactive. Furthermore, partial removal of α(1→2)-Man*p* branching may promote TLR2 activation, but this is only the case for Cg-LAM.

## Discussion

Apart from sharing a similar cell wall architecture, *C. glutamicum* possesses similar genetic loci responsible for cell wall biosynthesis like *M. tuberculosis*. As a model, it has proven extremely useful in the study of essential genetic elements of *M. tuberculosis* (Alderwick *et al*., 2006a,b;). In particular, it has enabled us to use it as a suitable model for the identification and functional study of mycobacterial genes involved in mycolic acid (Gande *et al*., 2004; 2007;), arabinogalactan (Alderwick *et al*., 2005; 2006b; 2007; Seidel *et al*., 2007a,b; Birch *et al*., 2008; 2009;) and LAM biosynthesis (Gibson *et al*., 2003; Mishra *et al*., 2007; 2008a,b; 2009; Tatituri *et al*., 2007a,b;). Previously, we reported the identification of enzymes involved in early and late stages of LM and LAM biosynthesis in *Corynebacterineae* (Mishra *et al*., 2007; 2008a,b; 2009;).

SDS-PAGE and MALDI-TOF-MS analysis of different lipoglycan species, including Cg-LM and Cg-LAM from *C. glutamicum*Δ*mptC* and *C. glutamicum*Δ*mptD*, suggested that there is a difference in a similar component of all these species. Two-dimensional thin-layer chromatography (2D-TLC) analysis supplemented with negative MALDI-TOF-MS of extracted glycolipids from mutants failed to show any differences in the PIM precursors (data not shown), and the presence of MptA and MptB in these mutants, negates the possibility of a change in the α(1→6) mannan backbone of these lipoglycans, leaving the only possibility of a change in α(1→2)-Man*p* branching, suggesting that MptC and MptD are involved in synthesis of these α(1→2)-Man*p* units in these lipoglycans. In addition, glycosyl linkage analysis revealed a reduction in the relative abundance of t-Man*p* and 2,6-Man*p* with a concomitant increase in the abundance of 6-Man*p* in Cg-LM and Cg-LAM from these strains. The cumulative chemical analysis of *C. glutamicum*Δ*mptC* and *C. glutamicum*Δ*mptD* agrees with the view that Cg-MptC and Cg-MptD are involved in synthesis of α(1→2)-Man*p* units in Cg-LM and Cg-LAM in *C. glutamicum*. This view was strengthened by the analysis of a *C. glutamicum*Δ*mptC*Δ*mptD* double mutant and the specificity of MptC and MptD in cross-complementation studies (Fig. S2). In summary, the data clarified the occurrence of residual t-α-Man*p*, α(1→2)-Man*p* and t-α-Ara*f* units in the Cg-MptC and Cg-MptD mutants, as the singular mutants only partially removed 2,6-Man*p* branching. The absence of these units in the *C. glutamicum*Δ*mptC*Δ*mptD* mutant provides evidence that these units are present as t-α-Man*p*-(α1→2)-Man*p* and t-α-Ara*f*-(α1→2)-Man*p* motifs (Fig. 9). Furthermore, specific *in vitro* assays utilizing a chemically synthesized nonasaccharide acceptor demonstrate that both Cg-*mptC* and Cg-*mptD* encode for α(1→2)-mannopyranosyltransferases, responsible for the synthesis of the α(1→2)-Man*p* units on the mannan backbone of LM and LAM. From the earlier studies with Rv2181 (Kaur *et al*., 2008), and the fact that Cg-MptC and Rv2181 are homologues, it is tempting to speculate that Cg-MptC adds α(1→2)-Man*p* residues to the proximal backbone of Cg-LM, or one could imply that Rv2181 *in vitro* has MptC function. A plasmid encoding *Rv2181* was used to transform the *C. glutamicum* mutants, and Rv2181 was able to restore the wild-type phenotype in only the *C. glutamicumΔmptC* mutant, and possessed α(1→2)-mannopyranosyltransferase activity in the defined *in vitro* acceptor assay reported in this study.

Whereas the LM/LAM backbone is potentially rather similar within the *Corynebacterineae*, as indicated also from the relatedness of the α(1→6)-mannopyranosyltransferases (Fig. 1), it is clear from structural, immunological and genetic evidence, that the more elaborated structures and decorations of these glycolipids are variable. Known structural variations include large arabinan domains in *M. tuberculosis* (Chatterjee *et al*., 1991; McNeil *et al*., 1994), Man-capping of arabinan domains in *M. tuberculosis* (Chatterjee *et al*., 1992) and their PI capping in *M. smegmatis* (Khoo *et al*., 1995). Accordingly, also immunological properties of LM/LAM are different (Briken *et al*., 2004). This variability is naturally reflected at the genetic level. An example is the capping GT-C transferase of *M. tuberculosis* encoded by *Rv1635c* (Appelmelk *et al*., 2008), which is not present in *C. glutamicum*. The fact that MptC in *C. glutamicum* is a fusion protein carrying a second domain with unknown function, also illustrates that mature LM and LAM glycolipids are species-specific and accordingly the corresponding biosynthesis genes more prone to rapid evolution. Duplication or fusion of genes may indicate that the types of enzymes involved in the later steps of LM and LAM synthesis are highly dynamic and of evolutionary change within the *Corynebacterineae* (Xie *et al*., 2003).

A key question in lipoglycan biosynthesis is the division of LM and LAM abundance in the cell wall. The separation of LM and LAM in *C. glutamicum*, biosynthetically probably occurs after the initial formation of the α(1→6) mannan backbone by MptB, followed by α(1→2)-Man*p* branching by MptC (or MptD). The product of these two enzymes would then be the preferred substrate for further α(1→6)-Man*p* elongation by MptA, followed by α(1→2)-Man*p* branching by MptD (or MptC) (Fig. 9). Additional GT-C glycosyltransferases remain to be characterized involved in further modification of the α(1→6)-mannan backbone. These include how the dimanoside (via MptE?) and Ara*f*-Man*p* side-chains are synthesized (AftE?) and are summarized in Fig. 9.

Both LAM and LM are immunomodulatory molecules (Briken *et al*., 2004). Whereas LAM has an important role during intracellular survival, for instance by preventing phagosome-lysosome fusion (Fratti *et al*., 2003), LM has strong pro-inflammatory activity through its ability to activate TLR2 (Vignal *et al*., 2003; Quesniaux *et al*., 2004; Gilleron *et al*., 2006). Previous research has demonstrated that the pro-inflammatory activity of LM is at least partly based upon its acylation status (Gilleron *et al*., 2006; Doz *et al*., 2007). PI-based LAM and LM species can differ in their degree of acylation, each carrying between one and four acyl chains. Investigation of the immune activating properties of differentially acylated LM species demonstrated that LM should at least be tri-acylated to be pro-inflammatory, with species carrying less acyl chains exhibiting immunosuppressive activity in a mechanism independent of TLR2 (Doz *et al*., 2007).

Here we investigated different Cg-LAM and LM species for their ability to activate TLR2. Similar to mycobacterial LAMs, wild-type Cg-LAM was found to be inactive (Fig. 8). This finding is consistent with previous observations that LAM species carrying only t-Ara*f* substitutions, such as LAMs isolated from *Rhodococcus ruber* and *Turicella otitidis*, are largely inactive (Nigou *et al*., 2008). Comparison of wild-type Cg-LAM to the LAMs isolated from the *C. glutamicum*Δ*mptC* and *C. glutamicum*Δ*mptD* mutant strains demonstrated that the mutant LAMs had gained the ability to activate TLR2 (Fig. 8). The explanation for this increased activity is currently not known. Interestingly, wild-type Cg-LM, like wild-type Cg-LAM, but unlike mycobacterial LM was found to be completely TLR2-inactive (Fig. 8). The reason for the discrepancy is unclear, but may be related to structural differences in their membrane anchors. In mycobacteria, LAMs and LMs share a common PI anchor, on which further assembly occurs to give mature LM and LAM. However, in *C. glutamicum*, although Cg-LAM contains a normal PI anchor, two different pools of Cg-LM (Cg-LM-A and Cg-LM-B) dominate the majority of lipoglycan composition of the corynebacterial cell wall (Tatituri *et al*., 2007a; Lea-Smith *et al*., 2008; Mishra *et al*., 2008b). The large majority of the Cg-LM species (∼95%), Cg-LM-B, contains an anchor based upon a di-acylated α-**D**-glucopyranosyluronic acid-(1→3)-glycerol moiety, and only a minor fraction contains the more classical anchor based on PI, Cg-LM-A (Mishra *et al*., 2008b). Despite the different lipid anchors, the mannan backbone structures of Cg-LM-A and Cg-LM-B are similar as revealed through GC-MS analysis of partially methylated alditol acetates (Mishra *et al*., 2008b). This means that the majority of Cg-LM species will contain a di-acylated GlcAGroAc_2_ anchor, which is a feature that has been demonstrated to be incompatible with activation of TLR2 (Doz *et al*., 2007).

Furthermore, a complete investigation of the structural and functional relationship of these lipoglycans during host–pathogen interaction requires the availability of mutants defective in their respective biosynthetic pathways. The availability of complete genome sequences of several mycobacteria and related actinomycetes, like *C. glutamicum*, and the development of novel tools for genetic manipulation has opened up these possibilities (Cole *et al*., 1998).

## Experimental procedures

### Bacterial strains, cell culture and growth conditions

*Corynebacterium glutamicum* and *E. coli* DH5*αmcr* were grown in LB broth at 30°C and 37°C respectively. For *C. glutamicum* complex medium BHI and minimal medium CGXII was used (Eggeling and Bott, 2005). Kanamycin and ampicillin were used at a concentration of 50 µg ml^−1^ for selection of recombinants. Samples for lipid analysis were prepared by harvesting cells at an OD of 10–15, followed by a saline wash and freeze drying. HEK293 cells transfected with TLR2 (Flo *et al*., 2002) were kept in DMEM (Invitrogen) containing 10% FCS, 100 U ml^−1^ penicillin, 100 µg ml^−1^ streptomycin, 0.5 mg ml^−1^ G418, 2 mM L-glutamine and 110 mg l^−1^ pyruvate.

### Construction of plasmids and strains

To delete Cg-*mptC* and Cg-*mptD* the deletion vectors pK19mobsacBΔCg-*mptC,* and pK19mobsacBΔCg-*mptD* were constructed. In each case cross-over PCR was used with genomic DNA as template and two different PCR's with primer pairs AB and CD (Table S1). The resulting PCR products served as template for primer pairs AD. The PCR product contained 12 nucleotides (nt) of the 3′ end of the respective gene together with genomic upstream sequences, and 36 nt of the 5′ end together with genomic downstream sequences. The inserts of all plasmids used in this work were confirmed by sequencing. Genes were deleted in by first introducing plasmids prepared from *E. coli via* electroporation into *C. glutamicum* and then selection for sucrose resistance in a procedure as described (Schäfer *et al*., 1994). Starting from *C. glutamicum*Δ*mptC* and using pK19mobsacBΔCg-*mptD* the double deletion mutant *C. glutamicum*Δ*mptC*Δ*mptD* was made. The starting strain used was the type strain *C. glutamicum* ATCC13032. Chromosomal deletions were confirmed using primer pairs AD, as well as the additional new primer pairs EF, hybridizing outside of the regions used for plasmid constructions.

To assay for complementation of chromosomal deletions four expression plasmids were used. To construct pEKEx2-Cg-*mptC* primer pairs Ex-2100-RBS-for and Ex-2100-rev were used to amplify the 5′ half of *NCgl2100*, the PCR product treated with SalI/EcoRI and ligated with similar treated pEKEx2. To construct pEKEx2-Cg-*mptD* primer pairs Ex-2097-RBS-for and Ex-2097-rev were used to amplify *NCgl2097*, the PCR product treated with SalI/EcoRI and ligated with similar treated pEKEx2. To construct pEKEx2-Mt-*Rv2181,* chromosomal DNA of *M. tuberculosis* served as template, and *Rv2181* was amplified using primer pairs Ex-Rv2181-for and Ex-Rv2181-rev, the former providing the sequence CTGCAG as a ribosome binding site. The amplified product was treated with PstI and EcoRI and ligated with pEKEx2. Plasmids were used to transform the respective mutants via electroporation to kanamycin resistance, and recombinants confirmed by plasmid preparation*.*

### Lipid extraction and analysis

Polar and apolar lipids were extracted as described by Dobson *et al*. (1985). Briefly, 6 g of dry *C. glutamicum* cells was treated in 220 ml of methanolic saline (20 ml 0.3% NaCl and 200 ml CH_3_OH) and 220 ml of petroleum ether for 2 h (Dobson *et al*., 1985). The suspension was centrifuged and the upper layer containing apolar lipids was separated. An additional 220 ml of petroleum ether was added, mixed and centrifuged as described above. The two upper petroleum ether fractions were combined and dried. For polar lipids, 260 ml CHCl_3_/CH_3_OH/0.3% NaCl (9:10:3, v/v/v) was added to the lower aqueous phase and stirred for 4 h. The mixture was filtered and the filter cake re-extracted twice with 85 ml of CHCl_3_/CH_3_OH/0.3% NaCl (5:10:4, v/v/v). Equal amounts of CHCl_3_ and 0.3% NaCl (145 ml each) were added to the combined filtrates and stirred for 1 h. The mixture was allowed to settle, and the lower layer containing the polar lipids recovered and dried. The polar lipid extract was examined by 2D-TLC on aluminium backed plates of silica gel 60 *F*_254_ (Merck 5554), using CHCl_3_/CH_3_OH/H_2_O (65:25:4, v/v/v) in the first direction and CHCl_3_/CH_3_COOH/CH_3_OH/H_2_O (40:25:3:6, v/v/v/v) in the second direction. The thin-layer chromatographic plates sprayed with the appropriate staining solution to detect the presence of lipids, glycolipids or phospholipids as described (Dobson *et al*., 1985).

### Extraction and purification of lipoglycans

Lipoglycans were extracted from delipidated cells as previously described (Ludwiczak *et al*., 2001). Briefly, cells were broken by sonication (MSE Soniprep 150, 12 micron amplitude, 60 s ON, 90 s OFF for 10 cycles, on ice) and the cell debris refluxed five times with 50% C_2_H_5_OH at 68°C, for 12 h intervals. The cell debris was removed by centrifugation and the supernatant containing lipoglycans, neutral glycans and proteins dried. This dried extract was then treated with hot phenol–H_2_O. The aqueous phase was dialysed and dried, followed by extensive treatments with α-amylase, DNase, RNase chymotrypsin and trypsin. This fraction was dialysed to remove the low MW break-down products formed after the enzyme treatment, thus yielding the crude lipoglycan fraction.

The crude lipoglycan extract was dried and resuspended in buffer A (50 mM ammonium acetate and 15% propan-1-ol) and subjected to Octyl Sepharose CL-4B chromatography (2.5 × 50 cm). The column (2.5 × 50 cm) was washed initially with four column volumes of buffer A to ensure removal of neutral glycans followed by buffer B (50 mM ammonium acetate and 50% propan-1-ol). The eluent was collected and concentrated to approximately 1 ml and precipitated using 5 ml of C_2_H_5_OH. The sample was dried using a Savant Speedvac and then resuspended in buffer C [0.2 M NaCl, 0.25% sodium deoxycholate (w/v), 1 mM EDTA and 10 mM Tris-HCl, pH 8] to a final concentration of 20 mg ml^−1^. The sample was gently mixed and left to incubate for 48 h at room temperature. The sample was then loaded onto a Sephacryl S-200 (2.5 cm × 50 cm) column previously equilibrated with buffer C. The sample was eluted with 400 ml of buffer C at a flow rate of 3 ml h^−1^, collecting 1.5 ml fractions. The fractions were monitored by SDS-PAGE using either a silver stain or a Pro-Q emerald glycoprotein stain and individual fractions pooled and dialysed extensively against buffer D (10 mM Tris-HCl, pH 8, 0.2 M NaCl, 1 mM EDTA) for 72 h with frequent changes of buffer. The samples were further dialysed against deionized water for 48 h with frequent changes of water, lyophilized and stored at −20°C.

### MALDI-TOF-MS and NMR analysis

The matrix used was 2,5-dihydroxybenzoic acid at a concentration of 10 µg µl^−1^, in a mixture of H_2_O/C_2_H_5_OH (1:1, v/v), 0.1% trifluoroacetic acid (TFA). Samples (0.5 µl) at a concentration of 10 µg µl^−1^ were mixed with 0.5 µl of the matrix solution. Analyses were performed on a Voyager DE-STR MALDI-TOF instrument (PerSeptive Biosystems, Framingham, MA, USA) using linear mode detection. Mass spectra were recorded in the negative mode using a 300 ns time delay with a grid voltage of 80% of full accelerating voltage (25 kV) and a guide wire voltage of 0.15%. The mass spectra were mass assigned using external calibration. NMR spectra of LAM samples were recorded on a Bruker DMX-500 equipped with a double-resonance (^1^H/X)-BBi z-gradient probe head. All samples were exchanged in D_2_O, with intermediate lyophilization, and then dissolved in 0.7 ml D_2_O and analysed at 313 K. The ^1^H and ^13^C NMR chemical shifts were referenced relative to internal acetone at 2.225 and 34.00 ppm respectively. All details concerning NMR sequences and the experimental procedures were described previously (Tatituri *et al*., 2007b).

### Glycosyl compositional and linkage analysis

Lipoglycans were hydrolysed using 250 µl of 2 M TFA at 120°C for 2 h, reduced with 100 µl of NaBD_4_ (10 mg ml^−1^ in C_2_H_5_OH:1 M NH_4_OH), and the resultant alditols per-*O*-acetylated using acetic anhydride (100 ml) at 120°C for 1 h, before examination by GC (Tatituri *et al*., 2007b). GC was performed using a Thermoquest Trace GC 2000 and a DB225 column (Supelco) with samples injected in the split-less mode. The oven was programmed to hold at an isothermal temperature of 275°C for a run time of 15 min. Glycosyl linkage analyses were performed as described previously (Tatituri *et al*., 2007b). Briefly, lipoglycan samples were per-*O*-methylated using dimethyl sulfinyl carbanion, hydrolysed using 2 M TFA, reduced using NaB_2_H_4_, and per-*O*-acetylated before analysis by GC-MS (Tatituri *et al*., 2007b). GC-MS was carried out on a Finnigan Polaris GCQ PlusTM using a BPX5 (Supleco) column. The injector temperature was set at 50°C, held for 1 min and then increased to 110°C at 20°C min^−1^. The oven was held at 110°C then ramped to 290°C at 8°C min^−1^ and held for 5 min to ensure all the products had eluted from the column. All the data were collected and analysed using Xcaliber (v.1.2) software.

### General methods for chemical synthesis of Acc-Man_9_ (compound 1)

Reactions were performed under a dry argon atmosphere. Whenever necessary, compounds and starting materials were dried by azeotropic removal of water with toluene under reduced pressure. The reactions were monitored by TLC on pre-coated silica gel (60F_254_) plates (0.25 mm) from E. Merck and visualized using UV light (254 nm) and/or heating after spraying with (NH_4_)_2_SO_4_ solution (150 g ammonium sulphate, 30 ml H_2_SO_4_, 750 ml H_2_O). Flash column chromatography was carried out on Isco Teledyne CombiFlash Rf200 chromatographic system using pre-packed columns. ^1^H and ^13^C NMR spectra were recorded at 400 MHz and 100 MHz, respectively, on a Varian 400-MR spectrometer. Coupling constants (*J*) are reported in Hz and chemical shifts (δ) in ppm relative to a residual solvent peak or an internal standard (TMS). The HR-MS spectra were recorded on a Agilent 6140 liquid chromatography mass spectrometry instrument.

### *p*-Tolyl 2,3-Di-*O*-benzoyl-4,5-*O*-benzylidene-1-thio-α-D-mannopyranoside (6)

Compound **5** (230 mg, 0.61 mmol) was dissolved in 5 ml of anhydrous pyridine, and benzoyl chloride (179 µl, 1.54 mmol) was added dropwise. The reaction mixture was stirred for 4 h at room temperature, poured into ice-water (15 ml) and extracted with CHCl_3_ (2 × 10 ml). The combined CHCl_3_ layers were washed with brine, dried over Na_2_SO_4_ and concentrated. Flash chromatographic purification of the crude product using cyclohexane/ethyl acetate as eluent gave pure **6** (335 mg, 94%) as a colourless solid (mp 66°C). *R_f_* = 0.44 (cyclohexane/ ethyl acetate, 2:1). ^1^H NMR (CDCl_3_, 400 MHz): δ 8.06 (2H, m, Ar), 7.92 (2H, m, Ar), 7.61 (1H, m, Ar), 7.46 (8H, m, Ar), 7.33 (4H, m, Ar), 7.15 (2H, d, *J* = 6.3 Hz, Ar), 5.95 (1H, dd, *J* = 0.9, 2.7 Hz, H-2), 5.82 (1H, dd, *J* = 2.7, 7.7 Hz, H-3), 5.68 (1H, s, CHPh), 5.59 (1H, d, *J* = 0.9 Hz, H-1), 4.64 (1H, ddd, *J* = 3.9, 6.9, 7.5, Hz, H-5), 4.39 (1H, dd, *J* = 7.2, 7.5 Hz, H-4), 4.33 (1H, dd, *J* = 3.6, 7.8 Hz, H-6a), 3.96 (1H, dd, *J* = 7.5, 7.8 Hz, H-6b), 2.34 (3H, s, CH_3_). ^13^C NMR (100 MHz, CDCl_3_): δ 165.40, 165.29 (2 × C = O), 138.52, 138.03, 133.54, 133.11, 133.00, 130.07, 129.98, 129.84, 129.79, 129.57, 129.48, 129.10, 128.59, 128.27, 128.25, 126.19 (Ar), 101.96 (CHPh), 87.31 (C-1), 76.95 (C-4), 72.44 (C-2), 69.22 (C-3), 68.61 (C-6), 65.21 (C-5), 21.17 (CH_3_). HR-MS: m/z 605.1604 [M + Na]^+^ calculated for C_34_H_30_O_7_SNa, found 605.1611.

### *p*-Tolyl 2,3-Di-*O*-benzoyl-6-*O*-benzyl-1-thio-α-D-mannopyranoside (7)

To an anhydrous CH_2_Cl_2_ (10 ml) solution of compound **6** (300 mg, 0.52 mmol) was added 200 mg of 4 Å molecular sieves under an atmosphere of argon and cooled to −78°C. Et_3_SiH (266 µl, 1.66 mmol) was added dropwise to the cooled reaction mixture followed by addition of triflic acid (115 µl, 1.30 mmol). The reaction mixture was then stirred at −78°C for 1 h. The reaction was quenched by the addition of triethylamine (0.25 ml) and CH_3_OH (0.5 ml), diluted with CH_2_Cl_2_ (20 ml), and washed with saturated aqueous NaHCO_3_ solution (10 ml), H_2_O (10 ml) and brine (10 ml). The organic layer was dried on Na_2_SO_4_, filtered and the filtrate concentrated to a syrup. Flash chromatographic purification of the crude product using cyclohexane/ethyl acetate as eluent gave pure **7** (241 mg, 80% yield) as colourless foam. *R_f_* = 0.38 (cyclohexane/ethyl acetate, 2:1). ^1^H NMR (CDCl_3_, 400 MHz): δ 8.03 (2H, m, Ar), 7.94 (2H, m, Ar), 7.58 (1H, m, Ar), 7.52 (1H, m, Ar), 7.43 (3H, m, Ar), 7.34 (8H, m, Ar), 7.08 (2H, d, *J* = 6.0 Hz, Ar), 5.85 (1H, dd, *J* = 0.9, 2.2 Hz, H-2), 5.61 (1H, d, *J* = 0.9 Hz, H-1), 5.57 (1H, dd, *J* = 2.2, 7.2 Hz, H-3), 4.69 (1H, d, *J* = 9.0 Hz, CH_2_Ph^a^), 4.59 (1H, d, *J* = 9.0 Hz, CH_2_Ph^b^), 4.53 (1H, m, H-5), 4.40 (1H, ddd, *J* = 2.7, 7.2, 7.5 Hz, H-4), 3.98 (1H, dd, *J* = 3.5, 7.8 Hz, H-6a), 3.89 (1H, dd, *J* = 2.7, 7.8 Hz, H-6b), 2.81 (1H, d, *J* = 3.0 Hz, 4-OH), 2.31 (3H, s, CH_3_). ^13^C NMR (100 MHz, CDCl_3_): δ 166.51, 165.37 (2 × C = O), 138.18, 138.04, 133.43, 133.38, 132.68, 129.93, 129.88, 129.85, 129.53, 129.41, 129.32, 128.53, 128.41, 128.39, 127.68, 127.60 (Ar), 86.42 (C-1), 73.76 (CH_2_Ph), 73.18 (C-3), 72.44 (C-5), 72.01 (C-2), 68.99 (C-6), 67.80 (C-4), 21.14 (CH_3_). HR-MS: m/z 607.1761 [M + Na]^+^ calculated for C_34_H_32_O_7_SNa, found 607.1757.

### *p*-Tolyl 4-*O*-Acetyl-2,3-di-*O*-benzoyl-6-*O*-benzyl-1-thio-α-D-mannopyranoside (4)

Compound **7** (200 mg, 0.34 mmol) was dissolved in 3 ml of dry pyridine, and acetic anhydride (49 µl, 0.51 mmol) was added under an argon atmosphere. The reaction mixture was stirred for 5 h at room temperature, poured into an ice-water mixture (10 ml) and extracted with CHCl_3_ (2 × 15 ml). The combined CHCl_3_ layers were washed with brine, dried over Na_2_SO_4_ and concentrated. Flash chromatographic purification of the crude product using cyclohexane/ethyl acetate as eluent gave pure **8** (202 mg, 94% yield) as colourless solid (m.p. 93.7°C). *R_f_* = 0.45 (cyclohexane/ethyl acetate, 2:1). ^1^H NMR (CDCl_3_, 400 MHz): δ 8.02 (2H, m, Ar), 7.91 (2H, m, Ar), 7.53 (2H, m, Ar), 7.45 (2H, m, Ar), 7.38 (2H, m, Ar), 7.32 (7H, m, Ar), 7.07 (2H, d, *J* = 6.0 Hz, Ar), 5.87 (1H, dd, *J* = 1.5, 2.2 Hz, H-2), 5.81 (1H, t, *J* = 7.5 Hz, H-4), 5.65 (1H, dd, *J* = 2.2, 7.3 Hz, H-3), 5.64 (1H, d, *J* = 1.5 Hz, H-1), 4.69 (1H, m, H-5), 4.63 (1H, d, *J* = 8.7 Hz, CH_2_Ph^a^), 4.51 (1H, d, *J* = 8.7 Hz, CH_2_Ph^b^), 3.75 (1H, dd, *J* = 3.4, 8.2 Hz, H-6a), 3.70 (1H, dd, *J* = 2.2, 8.2 Hz, H-6b), 2.30 (3H, s, PhCH_3_), 1.93 (3H, s, COCH_3_). ^13^C NMR (100 MHz, CDCl_3_): δ 169.76 (COCH_3_), 165.47, 165.40 (2 × COPh), 138.28, 137.99, 133.42, 133.34, 132.74, 129.94, 129.91, 129.81, 129.31, 129.20, 129.12, 128.53, 128.46, 128.33, 127.80, 127.60 (Ar), 86.24 (C-1), 73.62 (CH_2_Ph), 71.88 (C-2), 70.95 (C-5), 70.68 (C-3), 68.93 (C-6), 66.85 (C-4), 21.14 (PhCH_3_), 20.76 (COCH_3_). HR-MS: m/z 649.1867 [M + Na]^+^ calculated for C_36_H_34_O_8_SNa, found 649.1852.

### 1-Azidoethyl 4-*O*-Acetyl-2,3-di-*O*-benzoyl-6-*O*-benzyl-α-D-mannopyranosyl-(1→6)-α-D-mannopyranosyl-(1→6)-α-D-mannopyranosyl-(1→6)-2,3,4-tri-*O*-benzoyl-α-D-mannopyranosyl-2,3,4-tri-*O*-benzoyl-α-D-mannopyranosyl-(1→6)-2,3,4-tri-*O*-benzoyl-α-D-mannopyranosyl-(1→6)-2,3,4-tri-*O*-benzoyl-α-D-mannopyranosyl-(1→6)-2,3,4-tri-*O*-benzoyl-α-D-mannopyranosyl-(1→6)-2,3,4-tri-*O*-benzoyl-α-D-mannopyranoside (3)

To a solution of thiooctasaccharide **2** (150 mg, 0.04 mmol) in dry CH_2_Cl_2_ (5 ml) were added the donor thioglycoside **4** (47 mg, 0.08 mmol) and 100 mg of 4 Å molecular sieves. The mixture was cooled to −20°C and NIS (11.0 mg, 0.048 mmol) and triflic acid (2 µl, 0.02 mmol) added and the reaction was stirred for 15 min at −20°C. The temperature of the reaction mixture was raised to room temperature and after 1 h, 5 ml of saturated aqueous NaHCO_3_ was added. The reaction mixtures were extracted with 2 × 10 ml of CHCl_3_ and the organic layer was washed with saturated aqueous sodium thiosulfate solution (5 ml). The organic layer was dried over Na_2_SO_4_ and concentrated to a syrup. Flash chromatographic purification of the crude product was performed using cyclohexane/ethyl acetate as eluent and gave pure nonasaccharide **3** (169 mg, 82% yield) as colourless solid (m.p. 202°C). *R_f_* = 0.43 (cyclohexane/ethyl acetate, 1:1). ^1^H NMR (CDCl_3_, 400 MHz): δ 8.16 (15H, m, Ar), 8.05 (18H, m, Ar), 7.87 (17H, m, Ar), 7.47 (50H, m, Ar), 7.26 (35H, m, Ar), 6.18 (8H, m), 6.02 (2H, m), 5.98 (7H, m), 5.85 (3H, m), 5.26 (1H, s), 5.22 (1H, d, *J* = 1.2 Hz), 4.96 (6H, m), 4.48 (1H, d, *J* = 11.9 Hz, CH_2_Ph^a^), 4.57 (1H, dd, *J* = 1.8, 7.5 Hz), 4.38 (1H, dd, *J* = 2.4, 7.2 Hz), 4.28 (1H, d, *J* = 11.9 Hz, CH_2_Ph^b^), 4.13 (8H, m), 3.86 (9H, m), 3.66 (1H, dd, *J* = 2.1, 8.4 Hz), 3.57 (4H, m), 3.37 (7H, m), 1.88 (3H, s, CH_3_). ^13^C NMR (100 MHz, CDCl_3_): δ 166.79 (COCH_3_), 165.72, 165.56, 165.54, 165.52, 165.45, 165.37, 165.35, 165.33, 165.25, 165.23, 165.11 (COPh), 133.72, 133.51, 133.44, 133.29, 133.05, 132.96, 130.06, 130.01, 129.86, 129.67, 129.48, 129.42, 129.40, 129.37, 129.28, 129.21, 129.18, 129.15, 129.05, 128.82, 128.78, 128.70, 128.60, 128.51, 128.38, 128.27 (Ar), 98.28, 98.09, 97.95, 97.87 (C-1's), 73.45 (OCH_2_Ph), 70.88, 70.61, 70.53, 70.39, 70.29, 70.25, 70.17, 69.90, 69.71, 69.63, 69.54, 69.47, 69.40, 69.30, 67.19, 67.12, 66.74, 66.41, 66.32, 66.24, 66.03, 65.96, 65.75, 50.48 (CH_2_N_3_), 20.81 (COCH_3_).

### 1-Azidoethyl 6-*O*-Benzyl-α-D-mannopyranosyl-(1→6)-2,3,4-tri-*O*-benzoyl-α-D-mannopyranosyl-(1→6)-α-D-mannopyranosyl-(1→6)-α-D-mannopyranosyl-α-D-mannopyranosyl-(1→6)-α-D-mannopyranosyl-(1→6)-α-D-mannopyranosyl-(1→6)-α-D-mannopyranosyl-(1→6)-α-D-mannopyranoside (1)

To an ice-cooled solution of compound **3** (150 mg, 0.03 mmol) in dry CH_2_Cl_2_–CH_3_OH (1:2, 10 ml) was added a sodium methoxide solution in CH_3_OH (25% w/v, 2.0 ml). The reaction mixture was allowed to stir overnight at room temperature and H^+^ Amberlite resin (200 mg) was added. It was filtered, solvent was evaporated and the residue was washed with CHCl_3_ (3 × 10 ml). An aqueous solution (2 ml) crude nonasaccharide was passed through a small column of Bio-Beads SM-4 (15 g, 20–50 mesh) and eluted with 0–60% CH_3_OH–H_2_O. The aqueous solution that remained after brief evaporation under vacuum was frozen and lyophilized to a colourless foam (19 mg, 35%). ^1^H NMR (CDCl_3_, 400 MHz): δ 7.68 (1H, m), Ar), 7.38 (4H, m, Ar), 4.89 (1H, s), 4.64 (1H, d, *J* = 11.6 Hz, OCH_2_Ph), 4.61 (1H, d, *J* = 11.7 Hz, OCH_2_Ph), 3.99–3.60 (63H, m), 2.68 (2H, m, CH_2_N_3_). ^13^C NMR (100 MHz, CDCl_3_): δ 138.04, 128.39, 128.37, 127.01 (Ar), 101.77, 101.04, 100.96, 100.72, 100.55 (C-1's), 74.48, 74.37, 73.32 (OCH_2_Ph), 73.18, 72.86, 72.77, 72.62, 72.56, 72.42, 72.04, 70.97, 68.84, 68.71, 68.56, 67.86, 67.45, 67.31, 67.00, 51.79 (CH_2_N_3_). HR-MS: m/z 1658.5548 [M + Na]^+^ calculated for C_63_H_101_N_3_O_46_Na, found 1658.5503.

### *In vitro*α(1→2)-mannopyranosyltransferase assay utilizing a novel nonasaccharide acceptor, Acc-Man_9_

*Corynebacterium glutamicum*, *C. glutamicum*Δ*mptC*, *C. glutamicum*Δ*mptD*, complemented strains, and a double mutant devoid of Man*p* branching activities (MptC and MptD) were used to characterize the enzymatic activity of Cg-MptC and Cg-MptD, and Mt-Rv2181. Membranes from all strains of *C. glutamicum* were prepared as described previously (Mishra *et al*., 2008a) and resuspended in 100 mM sodium acetate (pH 6.0), containing 5 mM β-mercaptoethanol and 7 mM MgCl_2_ (Buffer E) to a final concentration of 10–15 mg ml^−1^. The chemically synthesized nonasaccharide acceptor, azidoethyl 6-*O*-benzyl-α-**D**-mannopyranosyl-(1→6)-[α-**D**-mannopyranosyl-(1→6)]_7_-**D**-mannopyranoside (Acc-Man_9_), and PP-[^14^C]-M [Gurcha *et al*., 2002; stored in CHCl_3_/CH_3_OH, 2:1, v/v], were aliquoted into 1.5 ml Eppendorf tubes to a final concentration of 2 mM and 250 000 cpm (0.305 Ci mmol^−1^), respectively, and dried under nitrogen. IgePal CA-630 (8 µl, Sigma Aldrich) was added and the tubes sonicated to resuspend the lipid linked components, and 1 mM ATP, 1 mM NADP, 5 mM MnCl_2_, and membrane protein (1 mg) added to a final volume of 100 µl. The reaction mixture incubated at 37°C for 2 h as described (Yokoyama and Ballou, 1989) and then terminated by the addition of 5 ml of 50% C_2_H_5_OH. The supernatant was recovered, dried and resuspended in 700 µl of water and loaded onto a 1 ml strong anion exchange SepPak cartridge (Supelco). The cartridge washed with H_2_O (5 ml), which was collected, dried and resuspended in 200 µl of H_2_O and 10% of the reaction product was quantified by scintillation counting using 5 ml of Ecolume (ICN Biomedicals, Costa Mesa, CA, USA) and the remaining material analysed by 15% SDS-PAGE-/autoradiography as described previously (Mishra *et al*., 2008a).

### Acc-Man_9_-[^14^C]-Man_x_ and selective cleavage by an α(1→2)-D-mannosidase

The extracted Acc-Man_9_-[^14^C]-Man_x_ products from the assays performed with *C. glutamicum* membranes were dried and subsequently incubated at 30°C for 24 h in 0.1 M acetate buffer, pH 5.0, with 5 mU of α(1→2)-**D**-mannosidase (Maras *et al*., 2000) as described previously (Ichishima *et al*., 1981; Yokoyama and Ballou, 1989). The reaction mixture was fractionated on a 50 ml Bio-Gel P-2 gel filtration column (30 cm × 1.5 cm; Bio-Rad) in H_2_O. The control Acc-Man_9_-[^14^C]-Man_x_ before α(1→2)-**D**-mannosidase treatment eluted form the Bio-Gel P-2 column at fractions 11–13, and digested products (release of [^14^C]Man*p*) was retained and eluted in later fractions 33–39 based on a [^14^C]-mannose standard (Cooper *et al*., 2002).

### Cell stimulation assays

Trypsine-released HEK293 cells were washed with and resuspended in culture medium at 1.11 × 10^6^ cells ml^−1^. Cells (1 × 10^5^, 90 µl) were transferred to a 96-well U-bottom plate (Greiner) and left for 2 h to let the cells re-adhere. After this, cells were stimulated (in triplicate) with 50 µg ml^−1^ wild-type or mutant Cg-LM and Cg-LAM (pretreated with 1% hydrogen peroxide for 96 h as described (Birch *et al*., 2010) in a final stimulation volume of 100 µl. Unstimulated cells served as a control. Culture supernatants were harvested after 24 h by centrifugation and stored at −80°C for cytokine measurements using enzyme-linked immunosorbent assay (ELISA) against human IL-8 (Invitrogen).

## References

[b1] Alderwick LJ, Radmacher E, Seidel M, Gande R, Hitchen PG, Morris HR (2005). Deletion of Cg-emb in *corynebacterianeae* leads to a novel truncated cell wall arabinogalactan, whereas inactivation of Cg-ubiA results in an arabinan-deficient mutant with a cell wall galactan core. J Biol Chem.

[b2] Alderwick LJ, Seidel M, Sahm H, Besra GS, Eggeling L (2006a). Identification of a novel arabinofuranosyltransferase (AftA) involved in cell wall arabinan biosynthesis in *Mycobacterium tuberculosis*. J Biol Chem.

[b3] Alderwick LJ, Dover LG, Seidel M, Gande R, Sahm H, Eggeling L (2006b). Arabinan-deficient mutants of *Corynebacterium glutamicum* and the consequent flux in decaprenylmonophosphoryl-D-arabinose metabolism. Glycobiology.

[b4] Alderwick LJ, Birch HL, Mishra AK, Eggeling L, Besra GS (2007). Structure, function and biosynthesis of the *Mycobacterium tuberculosis* cell wall: arabinogalactan and lipoarabinomannan assembly with a view to discovering new drug targets. Biochem Soc Trans.

[b5] Appelmelk BJ, den Dunnen J, Driessen NN, Ummels R, Pak M, Nigou J (2008). The mannose cap of mycobacterial lipoarabinomannan does not dominate the *Mycobacterium*-host interaction. Cell Microbiol.

[b6] Aqueel MS, Pathak V, Pathak AK (2008). Concise assembly of linear α(1**→**6)-linked octamannan fluorescent probe. Tetrahedron Lett.

[b7] Besra GS, Brennan PJ (1997). The mycobacterial cell wall: biosynthesis of arabinogalactan and lipoarabinomannan. Biochem Soc Trans.

[b8] Besra GS, Khoo KH, McNeil MR, Dell A, Morris HR, Brennan PJ (1995). A new interpretation of the structure of the mycolyl-arabinogalactan complex of *Mycobacterium tuberculosis* as revealed through characterization of oligoglycosylalditol fragments by fast-atom bombardment mass spectrometry and 1H nuclear magnetic resonance spectroscopy. Biochemistry.

[b9] Besra GS, Morehouse CB, Rittner CM, Waechter CJ, Brennan PJ (1997). Biosynthesis of mycobacterial lipoarabinomannan. J Biol Chem.

[b10] Birch HL, Alderwick LJ, Bhatt A, Rittmann D, Krumbach K, Singh A (2008). Biosynthesis of mycobacterial arabinogalactan: identification of a novel α(1**→**3) arabinofuranosyltransferase. Mol Microbiol.

[b11] Birch HL, Alderwick LJ, Rittmann D, Krumbach K, Etterich H, Grzegorzewicz A (2009). Identification of a terminal rhamnopyranosyltransferase (RptA) involved in *Corynebacterium glutamicum* cell wall biosynthesis. J Bacteriol.

[b12] Birch H, Alderwick LJ, Appelmelk BJ, Maaskant J, Bhatt A, Singh A (2010). A truncated lipoglycan from mycobacteria with altered immunological properties. Proc Natl Acad Sci USA.

[b13] Bloom BR, Murray CJ (1992). Tuberculosis: commentary on a reemergent killer. Science.

[b14] Brennan PJ (2003). Structure, function, and biogenesis of the cell wall of *Mycobacterium tuberculosis*. Tuberculosis (Edinb).

[b15] Brennan PJ, Ballou CE (1967). Biosynthesis of mannophosphoinositides by *Mycobacterium phlei*. The family of dimannophosphoinositides. J Biol Chem.

[b16] Brennan PJ, Ballou CE (1968). Biosynthesis of mannophosphoinositides by *Mycobacterium phlei*. Enzymatic acylation of the dimannophosphoinositides. J Biol Chem.

[b17] Brennan PJ, Nikaido H (1995). The envelope of mycobacteria. Annu Rev Biochem.

[b18] Briken V, Porcelli SA, Besra GS, Kremer L (2004). Mycobacterial lipoarabinomannan and related lipoglycans: from biogenesis to modulation of the immune response. Mol Microbiol.

[b19] Chatterjee D, Bozic CM, McNeil M, Brennan PJ (1991). Structural features of the arabinan component of the lipoarabinomannan of *Mycobacterium tuberculosis*. J Biol Chem.

[b20] Chatterjee D, Lowell K, Rivoire B, McNeil MR, Brennan PJ (1992). Lipoarabinomannan of *Mycobacterium tuberculosis*. Capping with mannosyl residues in some strains. J Biol Chem.

[b21] Cole ST, Brosch R, Parkhill J, Garnier T, Churcher C, Harris D (1998). Deciphering the biology of *Mycobacterium tuberculosis* from the complete genome sequence. Nature.

[b22] Cooper HN, Gurcha SS, Nigou J, Brennan PJ, Belisle JT, Besra GS (2002). Characterization of mycobacterial protein glycosyltransferase activity using synthetic peptide acceptors in a cell-free assay. Glycobiology.

[b23] Cumpstey I, Chayajarus K, Fairbanks AJ, Redgrave AJ, Seward CMP (2004). Allyl protecting group mediated intramolecular aglycon delivery: optimisation of mixed acetal formation and mechanistic investigation. Tetrahedron Asymmetry.

[b24] Daffe M, Brennan PJ, McNeil M (1990). Predominant structural features of the cell wall arabinogalactan of *Mycobacterium tuberculosis* as revealed through characterization of oligoglycosyl alditol fragments by gas chromatography/mass spectrometry and by 1H and 13C NMR analyses. J Biol Chem.

[b25] Dobson G, Minnikin DE, Minnikin SM, Parlett JH, Goodfellow M, Ridell M, Goodfellow M, Minnikin DE (1985). Systematic analysis of complex mycobacterial lipids. Chemical Methods in Bacterial Systematics.

[b26] Dover LG, Cerdeno-Tarraga AM, Pallen MJ, Parkhill J, Besra GS (2004). Comparative cell wall core biosynthesis in the mycolated pathogens, *Mycobacterium tuberculosis* and *Corynebacterium diphtheriae*. FEMS Microbiol Rev.

[b27] Doz E, Rose S, Nigou J, Gilleron M, Puzo G, Erard F (2007). Acylation determines the toll-like receptor (TLR)-dependent positive versus TLR2-, mannose receptor- and SIGNR1-independent negative regulation of pro-inflammatory cytokines by mycobacterial lipomannan. J Biol Chem.

[b28] Eggeling L, Bott M (2005). Handbook of Corynebacterium Glutamicum.

[b29] Flo TH, Ryan L, Latz E, Takeuchi O, Monks BG, Lien E (2002). Involvement of toll-like receptor (TLR) 2 and TLR4 in cell activation by mannuronic acid polymers. J Biol Chem.

[b30] Fratti RA, Chua J, Vergne I, Deretic V (2003). *Mycobacterium tuberculosis* glycosylated phosphatidylinositol causes phagosome maturation arrest. Proc Natl Acad Sci USA.

[b31] Gande R, Gibson KJ, Brown AK, Krumbach K, Dover LG, Sahm H (2004). Acyl-CoA carboxylases (accD2 and accD3), together with a unique polyketide synthase (Cg-pks), are key to mycolic acid biosynthesis in *Corynebacterianeae* such as *Corynebacterium glutamicum* and *Mycobacterium tuberculosis*. J Biol Chem.

[b32] Gande R, Dover LG, Krumbach K, Besra GS, Sahm H, Oikawa T (2007). The two carboxylases of *Corynebacterium glutamicum* essential for fatty acid and mycolic acid synthesis. J Bacteriol.

[b33] Geurtsen J, Chedammi S, Mesters J, Cot M, Driessen NN, Sambou T (2009). Identification of mycobacterial alpha-glucan as a novel ligand for DC-SIGN: involvement of mycobacterial capsular polysaccharides in host immune modulation. J Immunol.

[b34] Gibson KJ, Eggeling L, Maughan WN, Krumbach K, Gurcha SS, Nigou J (2003). Disruption of Cg-Ppm1, a polyprenyl monophosphomannose synthase, and the generation of lipoglycan-less mutants in *Corynebacterium glutamicum*. J Biol Chem.

[b35] Gilleron M, Nigou J, Nicolle D, Quesniaux V, Puzo G (2006). The acylation state of mycobacterial lipomannans modulates innate immunity response through toll-like receptor 2. Chem Biol.

[b36] Guerardel Y, Maes E, Elass E, Leroy Y, Timmerman P, Besra GS (2002). Structural study of lipomannan and lipoarabinomannan from *Mycobacterium chelonae*. Presence of unusual components with α1,3-mannopyranose side chains. J Biol Chem.

[b37] Gurcha SS, Baulard AR, Kremer L, Locht C, Moody DB, Muhlecker W (2002). Ppm1, a novel polyprenol monophosphomannose synthase from *Mycobacterium tuberculosis*. Biochem J.

[b38] Hill DL, Ballou CE (1966). Biosynthesis of mannophospholipids by *Mycobacterium phlei*. J Biol Chem.

[b39] Ichishima E, Arai M, Shigematsu Y, Kumagai H, Sumida-Tanaka R (1981). Purification of an acidic alpha-D-mannosidase from *Aspergillus saitoi* and specific cleavage of 1,2-α-D-mannosidic linkage in yeast mannan. Biochim Biophys Acta.

[b40] Kaur D, Berg S, Dinadayala P, Gicquel B, Chatterjee D, McNeil MR (2006). Biosynthesis of mycobacterial lipoarabinomannan: role of a branching mannosyltransferase. Proc Natl Acad Sci USA.

[b41] Kaur D, McNeil MR, Khoo KH, Chatterjee D, Crick DC, Jackson M (2007). New insights into the biosynthesis of mycobacterial lipomannan arising from deletion of a conserved gene. J Biol Chem.

[b42] Kaur D, Obregon-Henao A, Pham H, Chatterjee D, Brennan PJ, Jackson M (2008). Lipoarabinomannan of Mycobacterium: mannose capping by a multifunctional terminal mannosyltransferase. Proc Natl Acad Sci USA.

[b43] Khoo KH, Dell A, Morris HR, Brennan PJ, Chatterjee D (1995). Inositol phosphate capping of the nonreducing termini of lipoarabinomannan from rapidly growing strains of Mycobacterium. J Biol Chem.

[b44] Kordulakova J, Gilleron M, Mikusova K, Puzo G, Brennan PJ, Gicquel B (2002). Definition of the first mannosylation step in phosphatidylinositol mannoside synthesis. PimA is essential for growth of mycobacteria. J Biol Chem.

[b45] Kordulakova J, Gilleron M, Puzo G, Brennan PJ, Gicquel B, Mikusova K (2003). Identification of the required acyltransferase step in the biosynthesis of the phosphatidylinositol mannosides of mycobacterium species. J Biol Chem.

[b46] Kremer L, Gurcha SS, Bifani P, Hitchen PG, Baulard A, Morris HR (2002). Characterization of a putative α-mannosyltransferase involved in phosphatidylinositol trimannoside biosynthesis in *Mycobacterium tuberculosis*. Biochem J.

[b47] Lea-Smith DJ, Martin KL, Pyke JS, Tull D, McConville MJ, Coppel RL (2008). Analysis of a new mannosyltransferase required for the synthesis of phosphatidylinositol mannosides and lipoarbinomannan reveals two lipomannan pools in *Corynebacterineae*. J Biol Chem.

[b48] Liu J, Mushegian A (2003). Three monophyletic superfamilies account for the majority of the known glycosyltransferases. Protein Sci.

[b49] Ludwiczak P, Brando T, Monsarrat B, Puzo G (2001). Structural characterization of *Mycobacterium tuberculosis* lipoarabinomannans by the combination of capillary electrophoresis and matrix-assisted laser desorption/ionization time-of-flight mass spectrometry. Anal Chem.

[b50] McNeil M, Daffe M, Brennan PJ (1990). Evidence for the nature of the link between the arabinogalactan and peptidoglycan of mycobacterial cell walls. J Biol Chem.

[b51] McNeil M, Daffe M, Brennan PJ (1991). Location of the mycolyl ester substituents in the cell walls of mycobacteria. J Biol Chem.

[b52] McNeil MR, Robuck KG, Harter M, Brennan PJ (1994). Enzymatic evidence for the presence of a critical terminal hexa-arabinoside in the cell walls of *Mycobacterium tuberculosis*. Glycobiology.

[b53] Maras M, Callewaert N, Piens K, Claeyssens M, Martinet W, Dewaele S (2000). Molecular cloning and enzymatic characterization of a *Trichoderma reesei* 1,2-α-D mannosidase. J Biotechnol.

[b54] Mishra AK, Alderwick LJ, Rittmann D, Tatituri RV, Nigou J, Gilleron M (2007). Identification of an α(1**→**6) mannopyranosyltransferase (MptA), involved in *Corynebacterium glutamicum* lipomanann biosynthesis, and identification of its orthologue in *Mycobacterium tuberculosis*. Mol Microbiol.

[b55] Mishra AK, Alderwick LJ, Rittmann D, Wang C, Bhatt A, Jacobs WR (2008a). Identification of a novel α(1**→**6) mannopyranosyltransferase MptB from *Corynebacterium glutamicum* by deletion of a conserved gene, NCgl1505, affords a lipomannan- and lipoarabinomannan-deficient mutant. Mol Microbiol.

[b56] Mishra AK, Klein C, Gurcha SS, Alderwick LJ, Babu P, Hitchen PG (2008b). Structural characterization and functional properties of a novel lipomannan variant isolated from a *Corynebacterium glutamicum* pimB' mutant. Antonie Van Leeuwenhoek.

[b57] Mishra AK, Batt S, Krumbach K, Eggeling L, Besra GS (2009). Characterization of the *Corynebacterium glutamicum* deltapimB' deltamgtA double deletion mutant and the role of *Mycobacterium tuberculosis* orthologues Rv2188c and Rv0557 in glycolipid biosynthesis. J Bacteriol.

[b58] Morita YS, Patterson JH, Billman-Jacobe H, McConville MJ (2004). Biosynthesis of mycobacterial phosphatidylinositol mannosides. Biochem J.

[b59] Morita YS, Sena CB, Waller RF, Kurokawa K, Sernee MF, Nakatani F (2006). PimE Is a polyprenol-phosphate-mannose-dependent mannosyltransferase that transfers the fifth mannose of phosphatidylinositol mannoside in Mycobacteria. J Biol Chem.

[b60] Morr M, Takeuchi O, Akira S, Simon MM, Mühlradt PF (2002). Differential recognition of structural details of bacterial lipopeptides by toll-like receptors. Eur J Immunol.

[b61] Nigou J, Vasselon T, Ray A, Constant P, Gilleron M, Besra GS (2008). Mannan chain length controls lipoglycans signalling via and binding to TLR2. J Immunol.

[b62] Quesniaux VJ, Nicolle DM, Torres D, Kremer L, Guérardel Y, Nigou J (2004). Toll-like receptor 2 (TLR2)-dependent-positive and TLR2-independent-negative regulation of proinflammatory cytokines by mycobacterial lipomannans. J Immunol.

[b63] Schäfer A, Tauch A, Jäger W, Kalinowski J, Thierbach G, Pühler A (1994). Small mobilizable multi-purpose cloning vectors derived from the *Escherichia coli* plasmids pK18 and pK19: selection of defined deletions in the chromosome of *Corynebacterium glutamicum*. Gene.

[b64] Seidel M, Alderwick LJ, Birch HL, Sahm H, Eggeling L, Besra GS (2007a). Identification of a novel arabinofuranosyltransferase AftB involved in a terminal step of cell wall arabinan biosynthesis in *Corynebacterianeae*, such as *Corynebacterium glutamicum* and *Mycobacterium tuberculosis*. J Biol Chem.

[b65] Seidel M, Alderwick LJ, Sahm H, Besra GS, Eggeling L (2007b). Topology and mutational analysis of the single Emb arabinofuranosyltransferase of *Corynebacterium glutamicum* as a model of Emb proteins of *Mycobacterium tuberculosis*. Glycobiology.

[b66] Sena CB, Fukuda T, Miyanagi K, Matsumoto S, Kobayashi K, Murakami Y (2010). Controlled expression of branch-forming mannosyltransferase is critical for mycobacterial lipoarabinomannan biosynthesis. J Biol Chem.

[b67] Skovierova H, Larrouy-Maumus G, Pham H, Belanova M, Barilone N, Dasgupta A (2010). Biosynthetic origin of the galactosamine substituent of arabinbogalactan in *Mycobacterium tuberculosis*. J Biol Chem.

[b68] Tatituri RV, Alderwick LJ, Mishra AK, Nigou J, Gilleron M, Krumbach K (2007a). Structural characterization of a partially arabinosylated lipoarabinomannan variant isolated from a *Corynebacterium glutamicum ubiA* mutant. Microbiology.

[b69] Tatituri RV, Illarionov PA, Dover LG, Nigou J, Gilleron M, Hitchen PG (2007b). Inactivation of *Corynebacterium glutamicum* NCgl0452 and the role of MgtA in the biosynthesis of a novel mannosylated glycolipid involved in lipomannan biosynthesis. J Biol Chem.

[b70] Vignal C, Guérardel Y, Kremer L, Masson M, Legrand D, Mazurier J (2003). Lipomannans, but not lipoarabinomannans, purified from *Mycobacterium chelonae* and *Mycobacterium kansasii* induce TNF-alpha and IL-8 secretion by a CD14-toll-like receptor 2-dependent mechanism. J Immunol.

[b71] Xie G, Keyhani NO, Bonner CA, Jensen RA (2003). Ancient origin of the tryptophan operon and the dynamics of evolutionary change. Microbiol Mol Biol Rev.

[b72] Yokoyama K, Ballou CE (1989). Synthesis of α(1**→**6)-mannooligosaccharides in *Mycobacterium smegmatis*. Function of beta-mannosylphosphoryldecaprenol as the mannosyl donor. J Biol Chem.

[b73] Zhang N, Torrelles JB, McNeil MR, Escuyer VE, Khoo KH, Brennan PJ, Chatterjee D (2003). The Emb proteins of mycobacteria direct arabinosylation of lipoarabinomannan and arabinogalactan *via* an N-terminal recognition region and a C-terminal synthetic region. Mol Microbiol.

