# Supplementary material for: Lipoarabinomannan biosynthesis in Corynebacterineae: the interplay of two α(1→2)-mannopyranosyltransferases MptC and MptD in mannan branching
Source: Mol Microbiol. 2011 Jun;80(5):1241–59. doi: 10.1111/j.1365-2958.2011.07640.x (PMC3123699; doi:10.1111/j.1365-2958.2011.07640.x)

SUPPLEMENTARY MATERIAL

Supplementary Table S1: Primers used for plasmid and strain construction. Restriction sites are in bold, and overhangs required for hybridisation in the second PCR in italics. Primers are given in their 5´to 3´direction.

Primer	Sequences
A_{mptC}	CGTT**AAGCT**TTGTGTGCTAACTGCGTAATACTCGCGAC
B_{mptC}	*CCCATCCACTAAACTTAAACA*TCACCGTGCATGAAAAATAGTGTATCCG
C_{mptC}	*TGTTTAAGTTTAGTGGATGGG*TGGTTACTGCCGTATTTAGTTGTG
D_{mptC}	GTTT**GGATCC**ATACCGTAGTCAAATGCAGAGTCTTGAGC
E_{mptC}	CATTTGTGCCAACAACACGTGGTCATATGCG
F_{mptC}	GCGGATGCGATCACCGTCAACGC
A_{mptD}	CGCT**TCTAGA**GGATCATTCCACAATTTCCTACCCTCAG
B_{mptD}	*CCCATCCACTAAACTTAAACA*ATGTGCTGTCTGGGGGTTC
C_{mptD}	*TGTTTAAGTTTAGTGGATGGG*TTGGGTCTTGGGCTGAAAG
D_{mptD}	GCGG**GAATTC**CAGGCGGAATCCACATTGAGTTC
E_{mptD}	GTGATCATCGCAGTCGCAGTTGCTGC
F_{mptD}	GCATGGGCCACGACAAGGTTCGC
Ex-2100 RBS for	GATTAT**GTCGAC**AAGGAGATATAGATGATGCACGGTGAAAAACTTG TGG
Ex-2100 rev	GATATT**GAATTC**TCAAATGAGCGCTTTGTTTTGATTG
Ex-2097 RBS for	GATTAT**GTCGAC**AAGGAGATATAGATGTTGACGAACCCCCAGACAG C
Ex-2097 rev	GATATT**GAATTC**CTATTTGGATATTTTGGGAAATGC
Ex-Rv2181-for	GTGCTGCAGAAGGAGATATAGATATGAATTCGCCCTTGGTGGTCGG G
Ex-Rv2181-rev	CACGGATCCTTAGACGGTCACGGTCAGGCTG

Supplementary Figure S1: Characterization of chromosomal deletions in three different *C. glutamicum* mutants. Chromosomal DNA was prepared from the wild-type (WT), the deletion mutant *C. glutamicum* Δ*mptD* (D2097), the deletion mutant *C. glutamicum* Δ*mptC* (D2100), and the double deletion mutant *C. glutamicum* Δ*mptC* Δ*mptD* (D2100D20970). This served as template for PCR amplifications with primers specific for *mptC* (Probe-2100), and *mptD* (Probe-2097). A "+" denotes amplification with the specific primer pair. On the right standards are given with their size in kb.

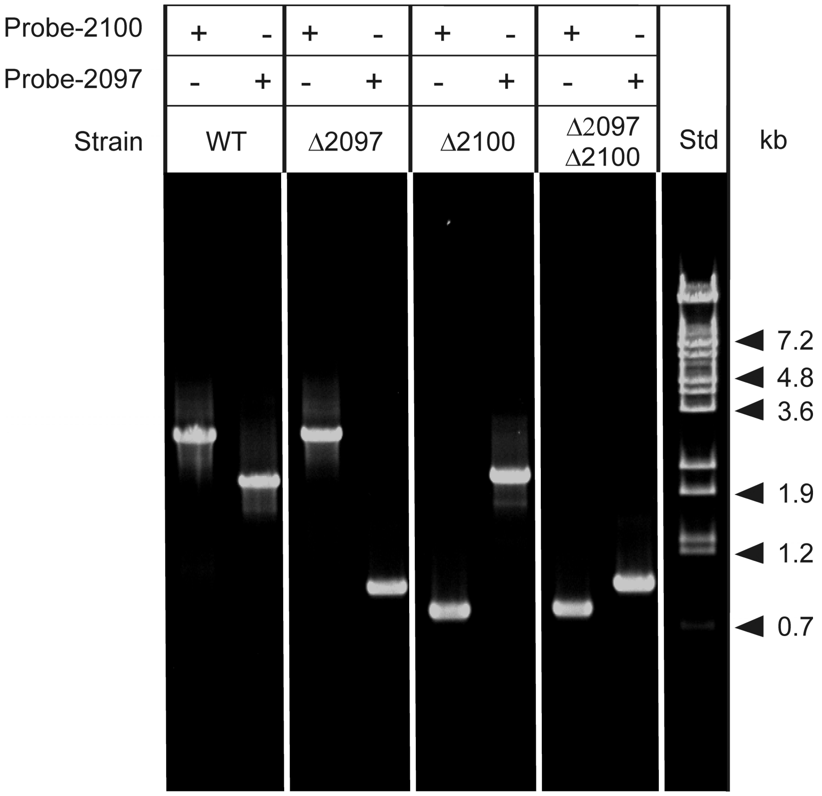

Supplementary Figure S2: Lipoglycan profiles of mutant and complemented strains of *C. glutamicum*. Lipoglycans were extracted from *C. glutamicum* Δ*mptC, C. glutamicum* Δ*mptC* pEKEx2-Cg-*mptD*, *C. glutamicum* Δ*mptD, C. glutamicum* Δ*mptD* pEKEx2-Cg-*mptC,* and analyzed using SDS-PAGE and visualized using a Pro-Q emerald glycoprotein stain (Invitrogen). Lipoglycan profiles are represented with standard molecular weight markers of glycoproteins of 180, 82, 42, and 18 kDa.

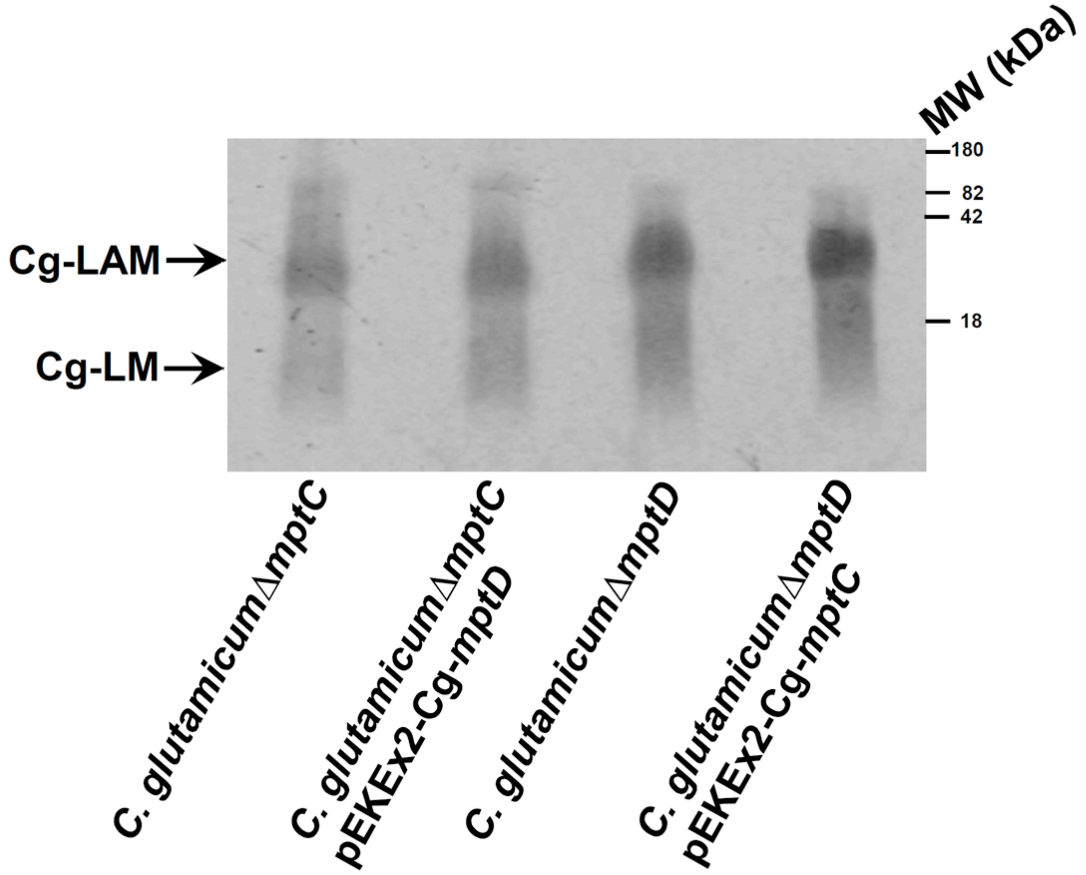

Supplementary Figure S3: Glycosyl compositional analysis of purified Cg-LM (A) and Cg-LAM (B) from different strains of *C. glutamicum*. Samples of individually purified lipoglycans were hydrolysed with 2M TFA, reduced and per-*O*-acetylated and subjected to GC analysis.

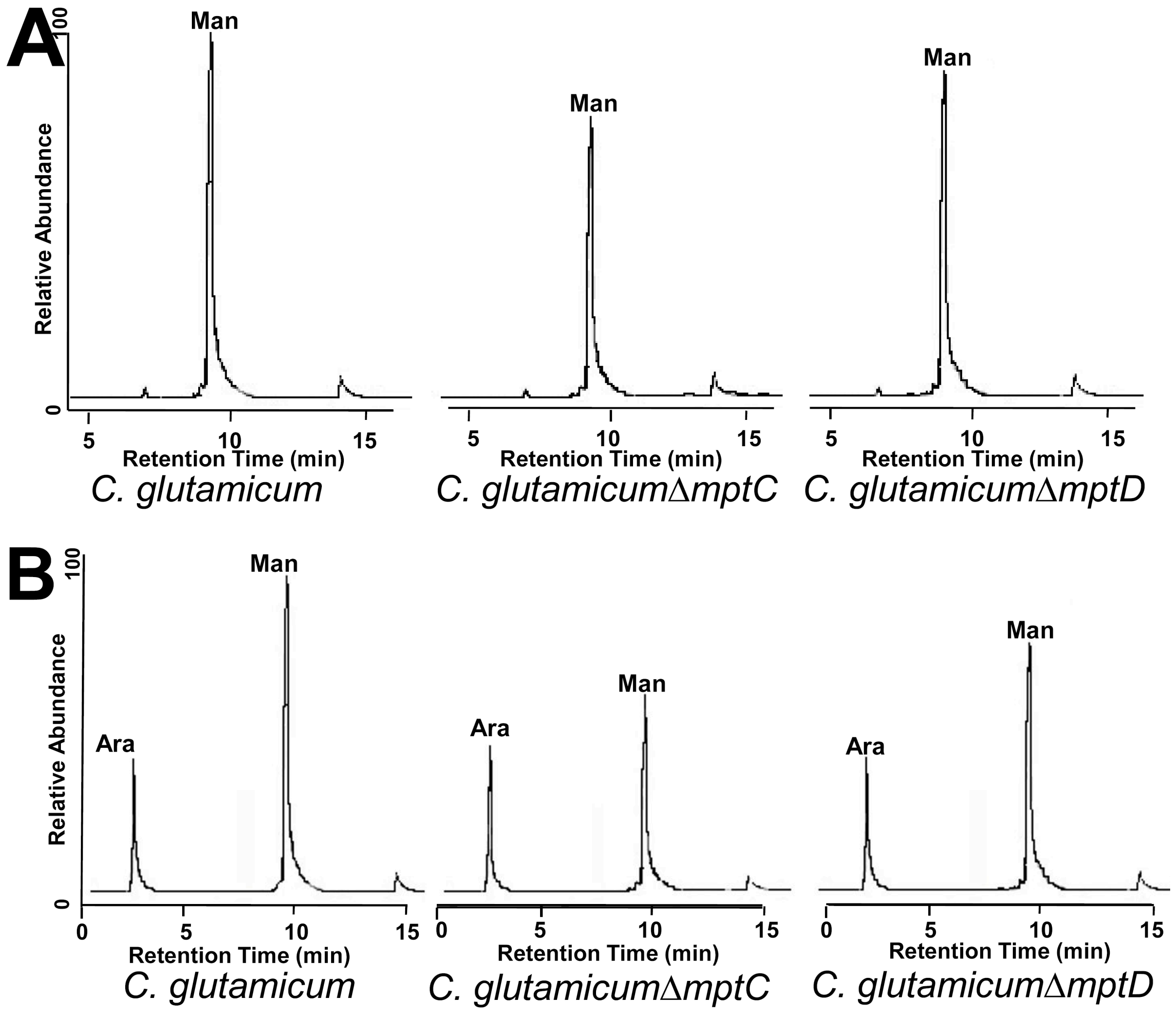

Supplementary Figure S4: GC-MS of per-*O*-methylated alditol acetate derivatives from Cg-LAM purified from *C. glutamicum∆mptC∆mptD* (A) and the identification of 2,3,4-tri-*O*-CH_3-1,5,6-tri-*O*-$COCH_3$-mannitol with the characteristic fragment alditol cleavage ions *m/z* 102, 118, 129, 162, 189, and 233 (B). GC-MS peaks marked (*) are non-carbohydrate impurities from the per-*O*-methylation and alditol acetate preparation procedures.

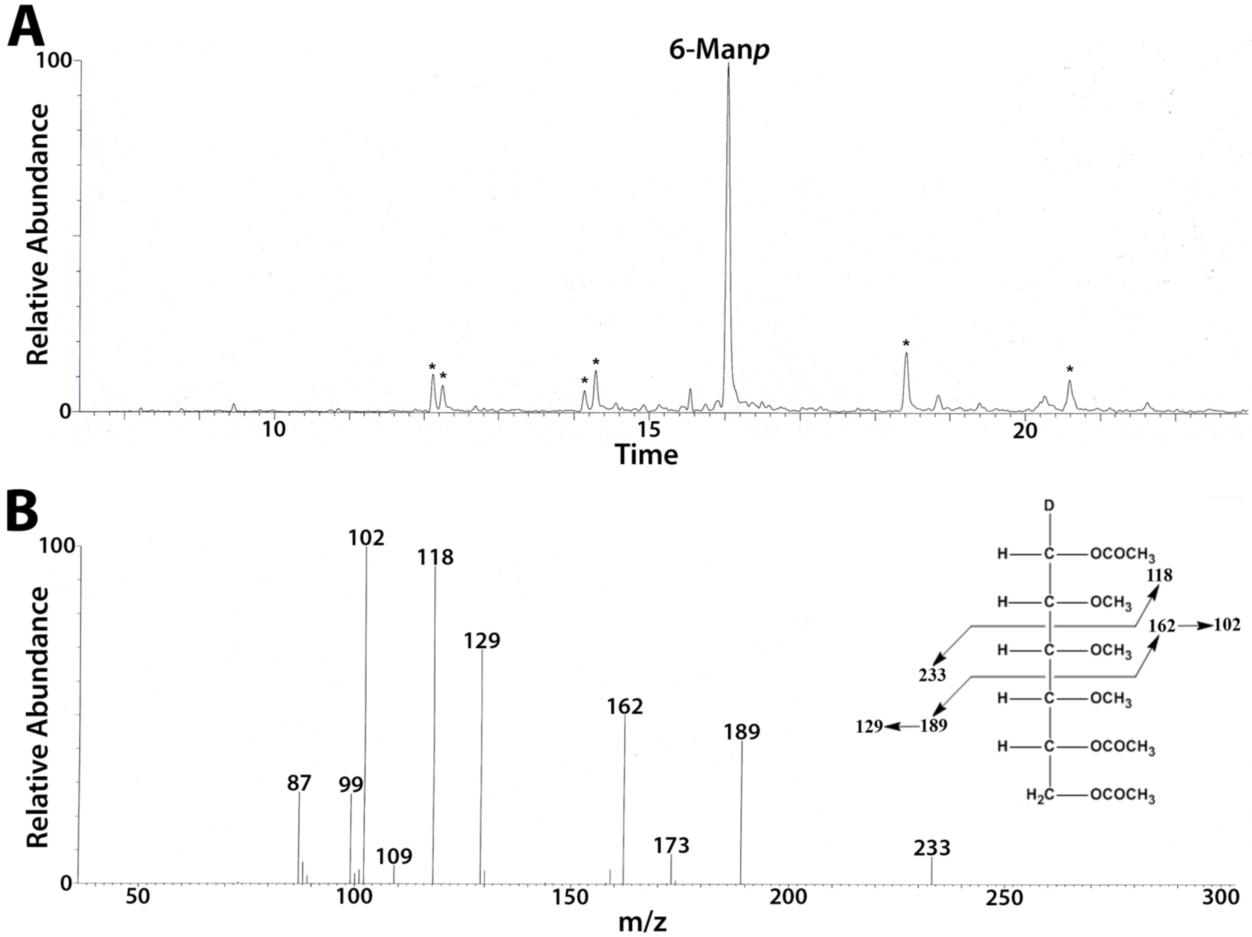

Supplement: Supplementary file 1 [file mmi0080-1241-SD1.pdf]
